# How do autoimmune diseases cluster in families? A systematic review and meta-analysis

**DOI:** 10.1186/1741-7015-11-73

**Published:** 2013-03-18

**Authors:** Jorge Cárdenas-Roldán, Adriana Rojas-Villarraga, Juan-Manuel Anaya

**Affiliations:** 1 Center for Autoimmune Diseases Research (CREA), School of Medicine and Health Sciences, Universidad del Rosario, Carrera 24 #63-C-69, Bogota, Colombia

**Keywords:** Autoimmune diseases, familial autoimmunity, aggregation, genetic epidemiology, autoimmune tautology

## Abstract

**Background:**

A primary characteristic of complex genetic diseases is that affected individuals tend to cluster in families (that is, familial aggregation). Aggregation of the same autoimmune condition, also referred to as familial autoimmune disease, has been extensively evaluated. However, aggregation of diverse autoimmune diseases, also known as familial autoimmunity, has been overlooked. Therefore, a systematic review and meta-analysis were performed aimed at gathering evidence about this topic.

**Methods:**

Familial autoimmunity was investigated in five major autoimmune diseases, namely, rheumatoid arthritis, systemic lupus erythematosus, autoimmune thyroid disease, multiple sclerosis and type 1 diabetes mellitus. Preferred Reporting Items for Systematic Reviews and Meta-Analysis (PRISMA) guidelines were followed. Articles were searched in Pubmed and Embase databases.

**Results:**

Out of a total of 61 articles, 44 were selected for final analysis. Familial autoimmunity was found in all the autoimmune diseases investigated. Aggregation of autoimmune thyroid disease, followed by systemic lupus erythematosus and rheumatoid arthritis, was the most encountered.

**Conclusions:**

Familial autoimmunity is a frequently seen condition. Further study of familial autoimmunity will help to decipher the common mechanisms of autoimmunity.

## Background

Autoimmune diseases (ADs) are chronic conditions initiated by the loss of immunological tolerance to self-antigens; they represent a heterogeneous group of disorders that afflict specific target organs or multiple organ systems [1]. The chronic nature of these diseases places a significant burden on the utilization of medical care, increases direct and indirect economic costs, and diminishes quality of life. The estimated incidence of ADs is approximately 80 per 100,000 person years and their prevalence could be well beyond 3% of the population [2]. Most of the ADs asymmetrically affect middle-aged women and are among the leading causes of death for this group of patients. Although the frequency of ADs varies between countries [3], various studies have shown that, for some ADs, associations are found across populations [4].

**Figure 1:**
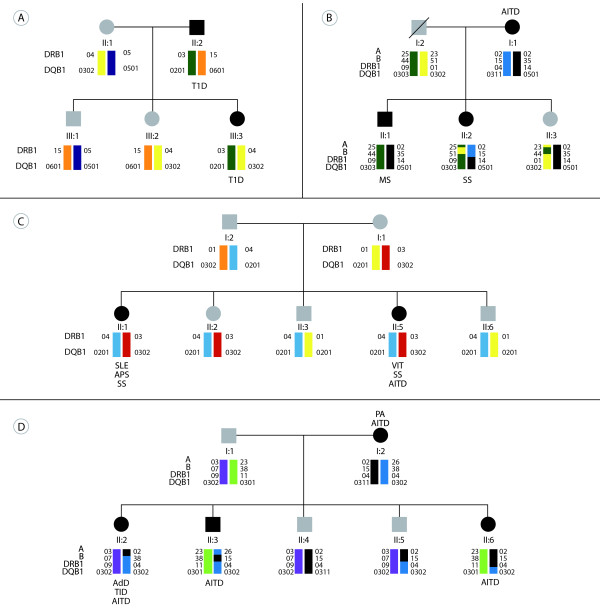
**How do autoimmune diseases cluster in families? A) **Familial autoimmune disease. This classical concept indicates the same AD in diverse FDRs. In this case, a proband and a FDR (that is, the father) present with T1D. **B) **Familial autoimmunity. This new concept corresponds to the presence of different ADs in a nuclear family. **C) **Multiple autoimmune syndrome. This condition refers to the presence of three or more autoimmune diseases in the same subject. In this case, two brothers met criteria for the syndrome. Moreover, this pedigree also meets criteria for familial autoimmunity. **D) **Polyglandular autoimmune syndrome type II. In this family, however, familial autoimmune disease and familial autoimmunity coexist. The results of HLA genes (that is, A, B, DRB1, DQB1) typing are shown in colors (by reverse dot blot using InnoLipa Kit). A suggestive linkage among the HLA loci is observed. In these diagrams, people are represented by symbols: circles for female and squares for male, and the bottom line represents the offspring of the couple above. Solid symbols represent affected individuals. Symbol with a diagonal line indicates deceased individual. AdD, Addison's disease; AITD, autoimmune thyroid disease; APS, antiphospholipid syndrome; FDRs, first degree relative; MS, multiple sclerosis; PA, pernicious anemia; SLE, systemic lupus erythematosus; SS, Sjögren's syndrome; T1D, type 1 diabetes; VIT, vitiligo.

ADs share several clinical signs and symptoms (that is, subphenotypes), physiopathological mechanisms, and genetic factors. These shared characteristics have been grouped under the term autoimmune tautology [5-10]. In clinical practice two conditions support this theory, namely, polyautoimmunity and familial autoimmunity, both of which are considered as being part of the 'kaleidoscope of autoimmunity' [11-14]. Whereas polyautoimmunity is the presence of two or more ADs in a single patient, familial autoimmunity occurs when relatives from a nuclear family present diverse ADs [9] (Figure 1). These conditions indicate that similar genetic, epigenetic, and environmental factors influence the development of ADs [7]. The best examples of polyautoimmunity are the multiple autoimmune syndrome (MAS), which occurs when a patient has three or more ADs [15,16], and the polyglandular autoimmune syndromes type II, III and IV [17], which are in fact MAS.

ADs do not begin at the moment they become clinically apparent but several years before. This implies that there is a chance to predict autoimmunity. Over the years, several risk factors have been associated with the onset of ADs. Among these the most widely studied are female gender [18], specific alleles at HLA and non-HLA loci [2,19] and some environmental agents [20,21]. In addition, the presence of auto antibodies may also predict specific clinical manifestations, disease severity and disease progression [22-27]. As reviewed by Tobon *et al*. [5] many auto antibodies have a predictive ability and they can be serologically evaluated long before the appearance of clinical disease. Thus, identification of these markers as well as a family history of autoimmunity and evaluation of their predictive value could be useful for personalized medicine.

A primary characteristic of complex diseases is that they are likely to aggregate in families (that is, familial aggregation, also referred to as recurrence risk or lambda, λ). The aggregation of a phenotype is observed when a disease occurs at a higher frequency in the relatives of an affected individual as compared with the frequency observed in the general population. Values of λ >1.0 indicate aggregation [9]. Aggregation of the same autoimmune condition, also referred to as familial autoimmune disease, has been extensively evaluated. However, aggregation of diverse autoimmune diseases, also known as familial autoimmunity, has been overlooked (Figure 1). Therefore, a systematic review and meta-analysis were performed aimed at gathering evidence about this topic.

## Methods

### Systematic review

A literature search was done even though 'familial autoimmunity' is not a Medical Subject Headings (MeSH) term. Nevertheless, the search was done in the electronic databases Medline and Embase, and included articles, from 1966 for the former and 1980 for the latter, up to June 2012. The search strategy was limited to humans and included the words '(familial OR clustering OR aggregation)' AND 'autoimmunity followed by each of the diseases we have focused on: 'multiple sclerosis,' 'diabetes mellitus, Type 1,' 'arthritis, rheumatoid' and 'lupus erythematosus, systemic' using MeSH terms and key words for 'autoimmune thyroid disease'. In order not to miss potentially eligible studies we used wild cards for the words familial, clustering and aggregation in the following manner: famil*, aggrega* and cluster*. No language restrictions were used. Articles were included if they fulfilled the following conditions: ADs diagnosis was carried out according to international criteria or through international classification of diseases, articles were published as full articles and, as mentioned earlier, if ADs in first degree relatives (FDRs) were different than in the proband. Studies were excluded if they only referred to autoantibody prevalence, if a clear cut distinction between diseases was not possible, if it was not possible to distinguish between probands and FDRs, if the studies were case reports, and if they dealt with a single family. Unpublished data were also excluded. Eligibility assessment was done by a primary reviewer who screened all titles and abstracts of publications. Retrieved articles were rejected if eligibility criteria were not met and a secondary reviewer was consulted in cases in which eligibility criteria were unclear. References from the articles that seemed to be relevant for our review were hand-searched. All articles were assessed according to the Oxford Centre for Evidence-based Medicine 2011 Levels of Evidence [28]. The search returned articles in which familial autoimmunity was assessed in other ADs and they were included. From each study we extracted data including total number of FDRs, numbers of FDRs affected, prevalence of ADs and, where possible, extraction of crude and adjusted measures of association, that is, odds ratio (OR) or risk ratio (RR). With the prevalences extracted, aggregation for different ADs across the five index diseases mentioned earlier was calculated by dividing the prevalence of a given AD in FDRs by the prevalence in the general population (λ_relatives_). We extracted data on prevalences from five reports [2,29-32]. Inclusion criteria for the meta-analyses were applied to publications that provided epidemiological data on risk factors, RR and OR with confidence intervals (CI), or that provided information that allowed us to calculate these data. If the study did not report the number of subjects in each group, either the RR or the OR with the CI, must have been reported in order for them to be included in the meta-analyses calculations.

In order to study aggregation, we determined worldwide prevalences of ADs from five studies mentioned earlier [2,29-32]. If a range was reported, we arbitrarily calculated the mean.

### Meta-analyses

Data were analyzed using the Comprehensive Meta-Analysis Version 2 program (Biostat, Englewood, NJ, 2004). Calculations were carried out for the whole group of articles depending on the binary data available for any AD: number of subjects and risk data (OR and RR with the corresponding 95% CI). Effect size was calculated based on studies that reported an OR with its respective 95% CI and from raw data given by case-control and cohort studies. If raw data from cohort studies were available, a second effect size was calculated with studies that only showed the RR and the respective 95% CI. Different study designs were used to compute the same effect size since the effect size had the same meaning in all studies and was comparable in relevant aspects. In order to perform the analyses, the association measures were transformed to log values, and then the results were converted back to ratio values for presentation. This approach prevented the omission of studies that used an alternative measure. Two types of meta-analyses were done in order to analyze autoimmunity as a trait. First, a given AD in FDRs was analyzed through all the studies regardless of the AD of the proband. The second type of meta-analysis analyzed ADs in FDRs through all the studies from a specific AD present in the proband.

**Figure 2:**
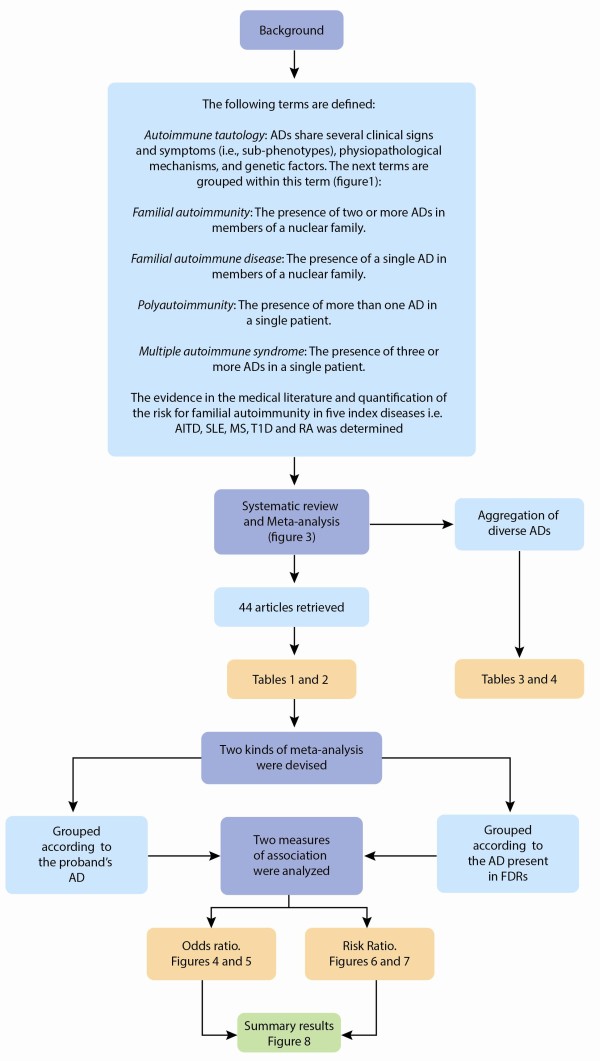
**Flow diagram of current study**. AITD, autoimmune thyroid disease; MS, multiple sclerosis; RA, rheumatoid arthritis; SLE, systemic lupus erythematosus; T1D, type 1 diabetes.

Additional meta-analyses were done for studies with complex data structures and non-cumulative results as the information for the different effects was not totally independent. This is the case for studies reporting multiple independent subgroups, that is, aggregation for son and daughter separately, within a study. A flow diagram of the current study is shown in Figure 2.

ORs were grouped by weighing individual ORs by the inverse of their variance. For each analysis, the final effect OR and 95% CI were obtained by means of the random effect model, which was preferred because it accepts distributions of true effect sizes rather than one true effect and assigns a more balanced weight to each study. It was also used because all the studies were considered to be unequal in terms of specific ADs.

Heterogeneity was calculated by means of Higgins's (*I^2^*) tests. The variance between studies was estimated by the DerSimonian and Laird method. The *I^2 ^*test showed the proportion of observed dispersion that was real rather than spurious and was expressed as a ratio ranging from 0% to 100%. *I^2 ^*values of 25%, 50%, and 75% were qualitatively classified as low, moderate, and high, respectively. Publication bias was determined using Funnel plots and Egger's regression asymmetry tests.

## Results

### Studies retrieved

**Figure 3:**
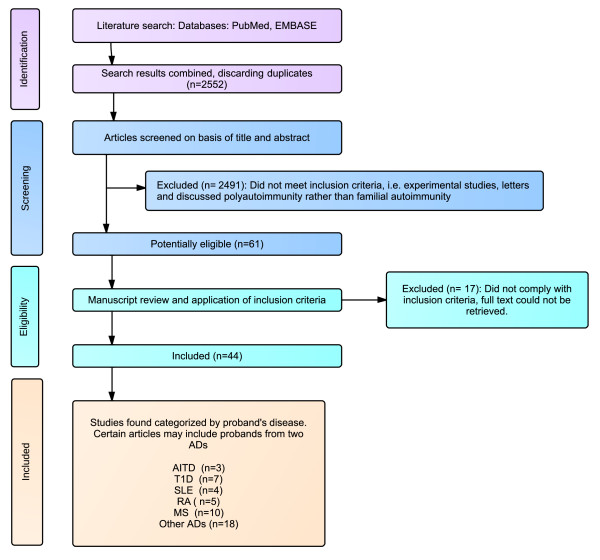
**Flowchart summarizing the search results**.

After discarding duplicates, the search in both databases retrieved 2,552 articles. In a first assessment we considered 61 articles to be eligible. In a second screening 17 of these articles were not eligible due to reporting inconsistencies, such as not distinguishing between probands and FDRs. As we did not identify other articles from the reference lists, only 44 articles met eligibility criteria [32-75]. Figure 3 and Table 1 summarize the search results. Although ankylosing spondylitis (AS) is considered an auto-inflammatory more than autoimmune disease [76], it was included in the results since it was found to aggregate in families. Most of the studies found lacked controls and had a small sample size, which is reflected in low grading according to the 2011 levels of evidence from the Oxford Centre for Evidence-based Medicine [28]. Detailed information is shown in Table 2.

**Table 1:** Significant associations of autoimmune diseases in first degree relatives.

Proband´s disease	Author	Disease in first degree relatives	References
Autoimmune thyroid disease	Boelaert *et al*.	AdD, CD, IBD, MG, MS, PA, RA, SLE, T1D, VIT	[33]
	Hemminki *et al*.	In parent: AdD, IIM, MG, PA, RA, T1D, UC	[34]
		In sibling: Discoid lupus erythematosus, localized SSc, T1D	
		In parent and sibling: SLE	
Type 1 diabetes	Bottazo *et al*.	AITD	[36]
	Anaya *et al*.	AITD	[37]
	Wagner *et al*.	AITD,CD, PSO, RA, VIT	[38]
	Hemminki *et al*.	In parent: AdD, AITD, AS, CD, PA, PBC, RA, SLE, UC, WG	[39]
		In sibling: AdD, AITD, CD	
		In parent and sibling: RA	
	Lebenthal *et al*.	AITD, CD	[41]
Systemic lupus erythematosus	Criswell *et al*.	AITD	[35]
	Alarcon-Segovia *et al*.	AITD, IIM, RA, SSc	[42]
	Priori *et al*.	AITD, RA, T1D, VIT	[43]
	Corporaal *et al*.	MS, RA	[44]
Rheumatoid arthritis	Lin *et al*.	AITD, T1D,	[45]
	Thomas *et al*.	T1D	[46]
	Hemminki *et al*.	In parent: AITD, AS, Localized SSc, PA, PSO, SLE, SS, SSc, WG	[47]
		In sibling: PSO, SLE	
	Jawaheer *et al*.	AITD	[48]
Multiple sclerosis	Barcellos *et al*.	AITD, PSO, RA, T1D	[32]
	Criswell *et al*.	PSO	[35]
	Broadley *et al*.	AITD	[49]
	Deretzi *et al*.	AITD, IBD, PSO, T1D	[50]
	Heinzlef *et al*.	AITD, RA, T1D, VIT	[51]
	Henderson *et al*.	AS	[52]
	Marrosu *et al*.	T1D	[53]
	Nielsen *et al*.	AdD, CrD, PAN,	[55]
Systemic sclerosis	Arora-Singh *et al*.	AITD, RA, SLE,	[57]
	Hudson *et al*.	AITD, PBC, RA, SLE, SS	[58]
	Koumakis *et al*.	AITD, RA, SLE, SS	[59]
Sjögren's syndrome	Reveille *et al*.	AITD, MS, SLE, SSc	[61]
	Anaya *et al*.	AITD, RA, SLE	[62]
Inflammatory bowel disease	Criswell *et al*.	MS	[35]
Ulcerative colitis	Hemminki *et al*.	In parent: AITD, AS, CrD, MS, PA, PAN, PSO, RA, SLE, T1D	[63]
		In sibling: AS, CrD	
		In parent and sibling: CrD, PSO	
Crohn's disease	Hemminki et al.	In parent: AS, PSO, UC	[63]
		In sibling: AS, UC	
		In parent and sibling: RA, UC	
		In twins: UC	
Vitiligo	Alkhateeb *et al*.	AdD, AITD, MG, PA, SLE, SSc	[64]
	Laberge *et al*.	AdD, AITD, PA, PSO, RA	[65]
	Zhang *et al*.	AA, PSO, RA	[66]
Juvenile rheumatoid arthritis	Prahalad *et al*.	AITD	[67]
	Huang *et al*.	AITD, AS, PSO, SLE	[68]
Juvenile lupus erythematosus	Huang *et al*.	AITD, AS, MG	[68]
Inflammatory idiopathic myositis	Ginn *et al*.	AITD, PA, PSO, RA, SS, T1D	[70]
Celiac disease	Petaros *et al*.	AITD, PSO, T1D	[72]
	Cataldo *et al*.	AITD, T1D	[73]
	Neuhausen *et al*.	JRA, T1D	[74]
Alopecia areata	Kakourou *et al*.	AITD, CD, PSO, T1D, VIT	[75]

AA, alopecia areata; AdD, Addison's disease; AS, ankylosing spondylitis; AITD, autoimmune thyroid disease; CD, celiac disease; CrD, Crohn's disease; IBD, inflammatory bowel disease; IIM, idiopathic inflammatory myositis; JDM, juvenile dermatomyositis; JRA, juvenile rheumatoid arthritis; JSLE, juvenile systemic lupus erythematosus; MAS, multiple autoimmune syndrome; MG, myasthenia gravis; MS, multiple sclerosis; PA, pernicious anemia; PAN, polyarteritis nodosa; PBC, primary biliary cirrhosis; PSO, psoriasis; RA, rheumatoid arthritis; SLE, systemic lupus erythematosus; SS, Sjögren's syndrome; SSc, systemic sclerosis; T1D, type 1 diabetes; UC, ulcerative colitis; VIT, vitiligo; WG, Wegener's granulomatosis. Note: Although AS is considered an auto-inflammatory more than autoimmune disease [76] we show the results obtained.

**Table 2:** Characteristics of the studies included.

Disease in probands	Author and year	Country	Study type	Total number of probands	Affected FDRs in probands	Total number FDRs	Total controls	Affected FDRs in controls	Total FDRs in controls	Oxford 2011 level of evidence	Observations
AITD											
	Boelaert 2010 [33]	UK	Cross sectional	3286	845	6572				3	
	Hemminki 2009 [34]	Sweden	Cohort	15,743	1,135	2,412				3	
	Criswell 2005 [35]	USA	Cross sectional	43	21	232				3	
T1D											
	Criswell 2005 [35]	USA	Cross sectional	10	6	232				3	
	Bottazzo 1978 [36]	UK	Cross sectional	116	4	257				3	
	Anaya 2006 [37]	Colombia	C&C	98	18	312	113	9	362	3	
	Wagner 2011 [38]	Spain	Cross sectional	12,973	97	1,279				3	Non diabetic siblings
				12,973	1,001	6,262					Diabetic siblings
	Hemminki 2009 [39]	Sweden	Cohort	21,168	1,913	5,195				3	
	Samuelsson 2004 [40]	Lithuania/Sweden	C&C	803	114		1,944	229		3	N = for controls and for FDRs is not specified
	Lebenthal 2011^a ^[41]	Israel	Cross sectional	121	57					3	Familial T1D patients
				226	43						Sporadic T1D patients.
											Numbers of FDRs are not given
SLE											
	Alarcón Segovia 2005 [42]	Latin america	Cross sectional	1,177	50					3	Total number of FDRs is not specified
	Priori 2003 [43]	Italy	C&C	154	39	759	140	12	776	3	
	Corporaal 2002 [44]	The Netherlands	Cross sectional	135	42	693				3	
	Criswell 2005 [35]	USA	Cross sectional	65	47	232				3	
RA											
	Criswell 2005 [35]	USA	Cross sectional	46	31	232				3	
	Lin 1998 [45]	USA	C&C	29	25	218	14	4	98	3	
	Thomas 1983 [46]	UK	C&C	295	19	2,081	307	8	2,299	3	
	Hemminki 2009 [47]	Sweden	Cohort	47,361	1,919	4,677				3	
	Jawaheer 2004 [48]	USA	C&C	1,097	10			83		3	N = for controls and for total FDRs are not specified
MS											
	Barcellos 2006 [32]	USA	Cross sectional	176	119	1,107				3	
	Criswell 2005 [35]	USA	Cross sectional	82	64	232				3	
	Broadley 2000 [49]	UK	C&C	571	879	3,949	375	879	3949	3	Individual numbers of FDRs of cases and controls are not given
	Deretzi 2010 [50]	Greece	C&C	891	410	3,112	355	96	1580	3	
	Heinzlef 2000 [51]	France	Cross sectional	357	33	1,971				4	
	Henderson 2000 [52]	Australia	C&C	157	53	722	222	1582	1138	3	
	Marrosu 2002 [53]	Italy	Cohort	1,090	53	5,480				3	
	Ramagopalan 2007 [54]	Canada	Cross sectional	5,031	786	30,259				3	
	Nielsen 2008 [55]	Denmark	Cohort	8,205	260	20,800				3	
	Laroni 2005 [56]	Italy	C&C	245	21	984	245	13	1,002	4	
SSc											
	Arora Singh 2010 [57]	USA		1,071	184	4,612				3	
	Hudson 2008 [58]	Canada	Cross sectional	719	260	715				3	
	Koumakis 2012 [59]	France	C&C	373	245	823	250	70	318	3	
	Frech 2010 [60]	USA	Nested C&C	1,037	95	4,629	10,370	417	49,312	3	Total number of FDRs, from controls, is not specified but ten controls were selected per proband
SS											
	Reveille 1984 [61]	USA	Cross sectional	98						3	Total number of FDRs and affected FDRs are not specified
	Anaya 2006 [62]	Colombia	C&C	101	56	876	124	33	857	3	
UC											
	Hemminki 2010 [63]	Sweden	Cohort	25,846	2,528	5,121				3	
CrD											
	Hemminki 2010 [63]	Sweden	Cohort	18,885	2,169	4,306				3	
IBD											
	Criswell 2005 [35]	USA	Cross sectional	7	8	232				3	
VIT											
	Alkhateeb 2003 [64]	USA/UK	Cross sectional	2,624	660	8,034				3	
	Laberge 2005 [65]	USA	Cross sectional	133	98	331				3	
	Zhang 2009 [66]	China	Cross sectional	5,601	340	18,705				3	
JRA											
	Prahalad 2002 [67]	USA	C&C	110	72	446	45	29	181	3	
	Huang 2004 [68]	Taiwan	Cross sectional	110	5					3	Total number of FDRs is not specified
JSLE											
	Huang 2004 [68]	Taiwan	Cross sectional	91	6					3	Total number of FDRs is not specified
	Walters 2012 [69]	USA	Cross sectional	69	23					3	Total number of FDRs is not specified
IIM											
	Ginn 1998 [70]	USA	C&C	21	33	151	21	7	143	3	
	Niewold 2011 [71]	USA	Cross sectional	304	30	1,224				3	
CD											
	Petaros 2002 [72]	Italy	C&C	125	18	373	125	4	352	3	
	Cataldo 2003 [73]	Italy	C&C	66	11	225	68	2	232	3	
	Neuhausen 2008 [74]	USA	Cross sectional	408	58	1,272				3	
AA											
	Kakouroru 2007 [75]	Greece	C&C	157			100			3	Total number of FDRs and affected FDRs are not specified
PSO											
	Criswell 2005 [35]	USA	Cross sectional	8	8	232				3	

^a^Numbers depicted represent families and not family members. Note: Only classical ADs are taken into account. Although some studies did not report the number of FDRs they specified other measures of association. See text for more details. AA, alopecia areata; AITD, autoimmune thyroid disease; C&C, case control; CD, celiac disease; CrD, Crohn's disease; IBD, inflammatory bowel disease; IIM, idiopathic inflammatory myositis; JRA, juvenile rheumatoid arthritis; JSLE, juvenile systemic lupus erythematosus; MS, multiple sclerosis; PSO, psoriasis; RA, rheumatoid arthritis; SLE, systemic lupus erythematosus; SS, Sjögren's syndrome; SSc, systemic sclerosis; T1D, type 1 diabetes; UC, ulcerative colitis; VIT, vitiligo.

### Autoimmune thyroid disease

Three articles assessed familial autoimmunity for autoimmune thyroid disease (AITD) [33-35]. AITD encompasses Graves' disease as well as Hashimoto's thyroiditis with the latter being the most common cause of acquired hypothyroidism [77]. Moreover, AITD is the most common AD [78]. Various studies have shown that AITD coexists with other ADs in the same subject [79,80] and it has also been shown that there is familial clustering of AITD in FDRs, particularly in female relatives [81]. Boelaert *et al*. [33] described familial autoimmunity among probands with Hashimoto's thyroiditis or Graves' disease. Both ADs were significantly associated with the presence of type 1 diabetes mellitus (T1D), rheumatoid arthritis (RA), pernicious anemia (PA), systemic lupus erythematosus (SLE), celiac disease (CD), vitiligo (VIT) and multiple sclerosis (MS). Only Graves' disease was associated with Addison's disease (AdD) and inflammatory bowel disease (IBD). Compared with the general population, familial autoimmunity in Graves' disease probands disclosed PA as the strongest association (RR: 14.1; 95% CI: 11.48 to 17.03), followed by RA (RR: 13.5; 95% CI: 12.32 to 14.86).

Hemminki *et al*. [34] assessed familial autoimmunity only in probands with Graves' disease from Sweden. To calculate familial risk within a large community based cohort they calculated standardized incidence ratios (SIR) as the ratio between the observed and the expected frequency for each disease. A value over one indicates an increased frequency of what is expected whereas a value below one indicates a decreased frequency. The analysis was stratified according to the FDR involved. For a single parent affected, Hashimoto's disease, PA, and RA were the only diseases significantly associated, having a SIR of 2.04, 1.82 and 1.48, respectively, thus showing an increased frequency of what is expected. Significant associations for singleton siblings were found for T1D, discoid lupus and localized scleroderma, having a SIR of 2.14, 6.03 and 6.62, respectively. If a parent and a sibling were affected with the same AD, the significant association was between Hashimoto's disease with a SIR of 37.41 and SLE with a SIR of 14.33 [34].

### Type 1 diabetes mellitus

The search returned seven articles about T1D probands [35-41]. AITD was responsible for the familial autoimmunity found in most of the articles [36,40], even when compared to control subjects [37]. Wagner *et al*. [38] replicated the results but also described the presence of CD, psoriasis (PSO), and VIT.

Hemminki *et al*. [39] also reported familial autoimmunity in probands with T1D. When a parent had AdD, the SIR for T1D in offspring was 2.41. It was 2.73 for CD, 1.83 for Graves' disease, 2.13 for Hashimoto's thyroiditis, 3.09 for PA, 3.63 for primary biliary cirrhosis (PBC), 2.12 for RA, 1.62 for SLE, 1.23 for ulcerative colitis (UC), and 1.23 for Wegener's granulomatosis (WG). Only the presence of AdD, CD or Graves' disease in singleton siblings was associated with T1D in probands. Likewise, when a parent and sibling had RA, the SIR for T1D was 5.34 [39].

### Systemic lupus erythematosus

Four articles assessed familial autoimmunity in SLE probands. Alarcon-Segovia *et al*. evaluated familial aggregation in the 'Grupo Latinoamericano de Estudio de Lupus' (GLADEL) [42]. They found that among all family members who had any AD, 6.7% had RA, 2% AITD and other ADs at a lesser frequency. In FDR (n = 114) with ADs, 28% (n = 32) had RA and 16% (n = 32) had AITD [42]. Likewise, an increased frequency of familial autoimmunity was found in SLE probands compared with population prevalence. Priori *et al*. [43] found an OR of 4.6 (95% CI 1.94 to 11.1) in a multivariate analysis of familial autoimmunity in FDR of SLE patients. They reported AITD as the most frequent disease with eight cases, followed by RA with five cases, VIT with three cases and T1D with two cases. PSO frequency was higher among non-autoimmune controls. Sjögren´s syndrome (SS) as well as AITD were described by Scofield *et al*. [82] while Corporaal *et al*. [44] found clustering of MS and RA.

### Rheumatoid arthritis

In RA, familial autoimmunity was ascertained in five articles, all of which linked AITD or T1D to AR. Lin *et al*. [45] showed an association with AITD in 7.8% of the probands and T1D in 2.8%. Thomas *et al*. [46] also reported T1D as the disease responsible for familial autoimmunity. In another study, Taneja *et al*. [83] stated that SLE, T1D, AITD, SS, PSO and systemic sclerosis (SSc) were found in families with RA. However, they included probands within this description, thus assessing and combining polyautoimmunity or MAS with familial autoimmunity. Walker *et al*. [84] found an excess risk for AITD in RA multicase families compared with the general population. However, this significance was lost when RA sufferers were withdrawn from the analysis. Jawaheer *et al*. [48] found the presence of AITD and other ADs in siblings but, compared with siblings of non-RA probands, the difference was not significant.

Hemminki *et al*. [47] also reported familial autoimmunity in probands with RA. Just as described above, when a parent had AS, the SIR for RA in offspring was 2.96. It was 2.25 for SS, 2.13 for SLE, 1.65 for SSc, 1.54 for AITD, 1.53 for PA, 1.36 for PSO and 1.34 for WG. When singleton siblings had PSO, the SIR for RA of the proband was 2.01 and 2.77 for SLE.

### Multiple sclerosis

In our search, MS was the AD with the most articles assessing familial autoimmunity with 10 articles found [32,35,49-56]. Some studies suggest that FDRs and other relatives of probands with MS could be at greater risk of ADs other than MS [32,35,49-55,57] while the studies done by Ramagopalan *et al*. [54] and Midgard *et al*. [85] do not support these findings. Although Annunziata *et al*. [86] found an association between MS and other ADs in first and second degree relatives, the results were not significant when compared to non-AD controls. Conversely, Alonso *et al*. [87] and Magaña *et al*. [88] found a significant association between MS and other ADs in relatives of any degree.

Using 265 families from the Multiple Autoimmune Disease Genetics Consortium (MADGC), Criswell *et al*. [35] compared the frequency of ADs in siblings of multiplex families stratified by seven ADs: AITD, RA, MS, SLE, T1D, IBD and PSO. These diseases were pre-specified given a variety of considerations. There was no evidence of familial autoimmunity except in the case of IBD patients in whose families MS was observed among FDRs (OR: 8.1; 95% CI: 1.77 to 37.0; *P *value = 0.018). However, selection bias was present as families selected for inclusion were not recruited in the same manner [35].

### Meta-analyses

**Figure 4:**
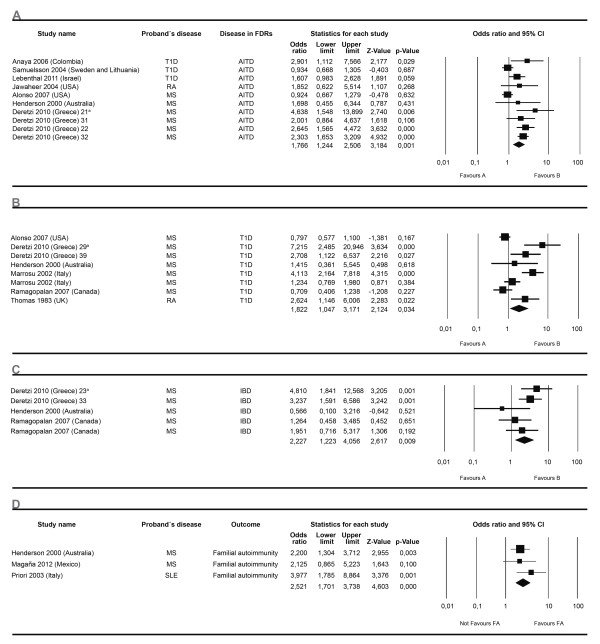
**Forest plots depicting odds ratios for specific autoimmune diseases in first degree relatives**. Familial autoimmunity has to be seen as a two way relationship depending on which member of the nuclear family is the proband. Therefore, grouping meta-analysis by the disease present in FDRs is equivalent to analyzing it by the disease present in the proband. The figure shows four different analyses. From top to bottom autoimmune thyroid disease (**A**), type 1 diabetes mellitus (**B**), inflammatory bowel disease (**C**) and familial autoimmunity (**D**) assessed as an outcome. The summary effect (random effect model) is depicted as a diamond at the bottom of each analysis. The lateral points of each diamond indicate confidence intervals for this estimate. ^a^Numbers represent different subgroups within the study.

**Figure 5:**
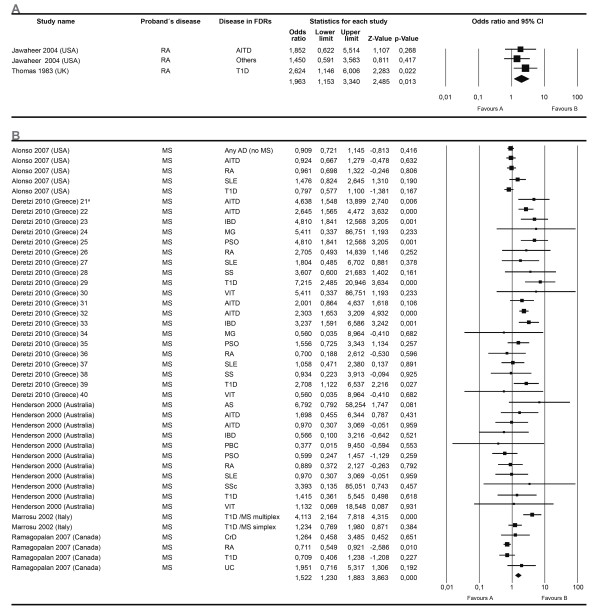
**Forest plots depicting odds ratios for familial autoimmunity**. The figure shows two different analyses. From top to bottom: (**A**) rheumatoid arthritis (RA), (**B**) multiple sclerosis (MS). Autoimmune diseases in first degree relatives through all the studies from a specific autoimmune disease present in the proband were analyzed. The summary effect (random effect model) is depicted as a diamond at the bottom of each analysis. The lateral points of each diamond indicate confidence intervals for this estimate. ^a^Numbers represent different subgroups within the study.

For the first effect size, OR, 13 meta-analyses were developed. Ten analyzed the proportion of a specific AD in FDRs independent of the AD present in the proband. Of these, three showed significant association: AITD, T1D and IBD. Three included an independent AD in FDRs in a specific AD of the proband, two of them showed significant associations: RA and MS. Figures 4 and 5 show the forest plots corresponding to six meta-analyses.

**Figure 6:**
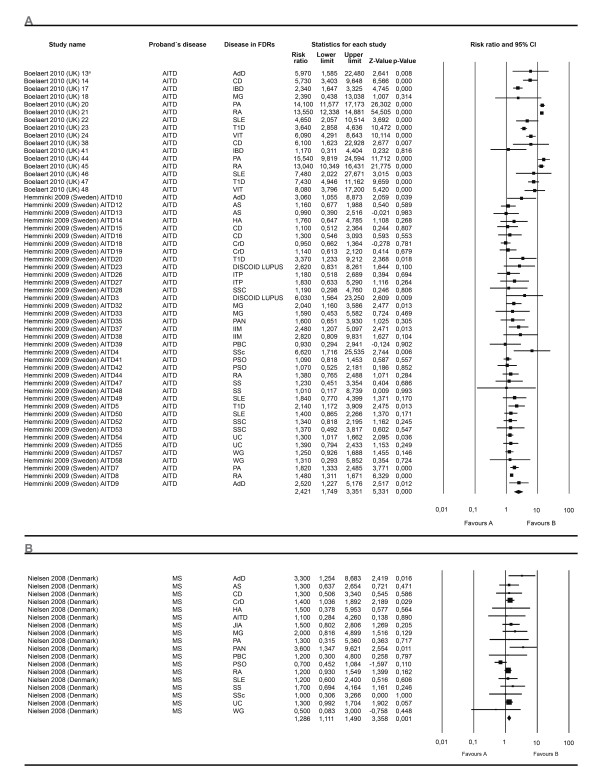
**Forest plots depicting risk ratios for familial autoimmunity in probands with AITD and MS**. The figure shows two different analyses. From top to bottom: autoimmune thyroid disease (**A**) and multiple sclerosis (**B**). The summary effect (random effect model) is depicted as a diamond at the bottom of each analysis. The lateral points of each diamond indicate confidence intervals for this estimate. ^a^Numbers in the study name represent different subgroups within the study. AITD, autoimmune thyroid disease; MS, multiple sclerosis.

**Figure 7:**
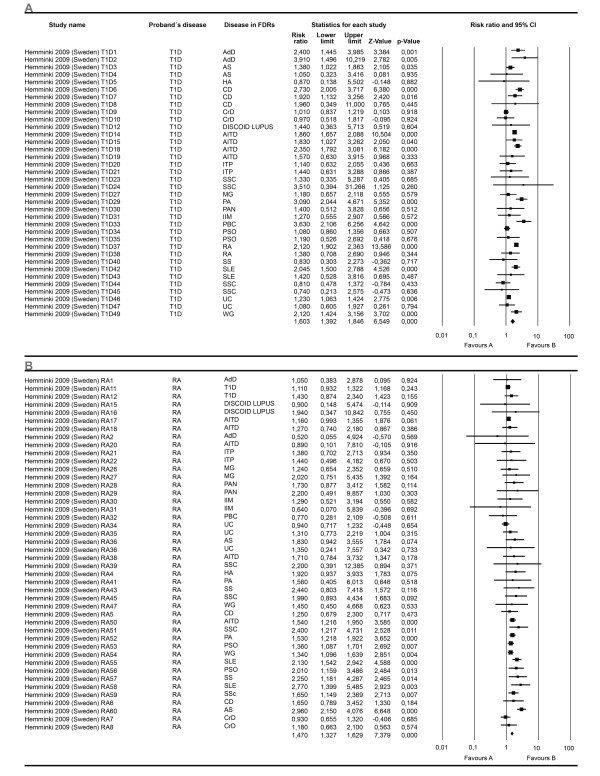
**Forest plots depicting risk ratios for familial autoimmunity in probands with T1D and RA**. The figure shows two different analyses. From top to bottom: type 1 diabetes mellitus (**A**) and rheumatoid arthritis (**B**). The summary effect (random effect model) is depicted as a diamond at the bottom of each analysis, the lateral points of which indicate confidence intervals for this estimate. ^a^Numbers in the study name represent different subgroups within the study. RA, rheumatoid arthritis; T1D, type 1 diabetes.

A second effect size was calculated based on data from studies showing RR data. Twenty eight meta-analyses were developed. Twenty three analyzed the proportion of a specific AD in the FDR through all the studies independent of any AD of the proband. Of these, nineteen showed significant association, the most relevant results being related to VIT, PA, RA and T1D. Additional results are shown in Additional file 1. Through all the studies, four additional analyses performed included any AD present in FDRs. All these analysis disclosed significant results. The ADs in the proband were AITD, MS, RA and T1D (Figures 6 and 7).

Evidence of significant publication bias was identified using the Egger test (*P*-value 2-tailed: <0.05) for two meta-analyses which included studies that reported OR with its respective 95% CI (T1D in FDR (*P*-value 2-tailed: 0.047) and MS in probands (*P*-value 2-tailed: 0.007)). One meta-analysis that reported RR data showed publication bias by the Egger test (AITD in probands (*P*-value 2-tailed: 0.008)) (Figure 6A). The corresponding funnel plot showing the standard error or the precision on the Y axis is shown in Additional file 2. Therefore, a second analysis was run in a search for publication bias. The classic fail-safe analysis indicated a number of missing studies that would give a *P*-value of >0.05. Begg and Mazumdar rank correlation was not significant and the trim and fill adjustment did not suggest a lower risk than the original analysis. Based on all the analyses for publication bias, we consider the impact of bias in the three meta-analyses to be trivial.

Familial autoimmunity as an outcome was also assessed in certain articles, particularly in MS and SLE probands (Figure 4D).

### Aggregation

Several studies retrieved only reported prevalences of ADs in FDRs. Aggregation, based on data from five studies mentioned earlier in Table 3[2,29-32], is shown in Table 4, which discloses information on calculated aggregation for diverse ADs, in AITD, T1D, SLE, RA and MS.

**Table 3:** Prevalence of specific autoimmune diseases^a^

Autoimmune disease	Prevalence (%)
Any autoimmune disease	3 to 8
Addison's disease	0.009
Alopecia areata	0.15
Ankylosing spondylitis	0.20
Autoimmune hepatitis	≤0.001
Celiac disease	0.75
CREST syndrome	≤0.001
Diabetes (type 1)	0.190
Graves' disease	1.200
Hashimoto's thyroiditis	0.800
Idiopathic thrombocytopenic purpura	≤0.001
Inflammatory bowel disease	0.450
Juvenile idiopathic arthritis	0.148
Multiple sclerosis	0.058
Myasthenia gravis	0.005
Pernicious anemia	0.150
Polymyositis/dermatomyositis	0.005
Primary biliary cirrhosis	0.004
Psoriasis	0.75
Rheumatoid arthritis	1
Sjögren's syndrome	0.3
Systemic lupus erythematosus	0.024
Systemic sclerosis (scleroderma)	0.004
Vitiligo	0.4
Wegener's granulomatosis	0.003

^a^Prevalences according to [2,29-32]. CREST, Calcinosis, Raynaud phenomenon, esophageal dysmotility, sclerodactyly, and telangiectasia.

**Table 4:** Aggregation of ADs.

	Disease in probands																						
	**T1D**					**AITD**		**SLE**			**RA**				**MS**								

**AD in FDRs**	**USA **[35]	**UK **[36]	**Colombia **[37]	**Spain **[38]	**Swed/Lith **[40]	**UK **[33]	**USA **[35]	**USA **[35]	**Latin America **[42]	**NL **[44]	**USA 1 **[35]	**USA 2 **[45]	**UK **[46]	**USA 3 **[48]	**USA1 **[32]	**USA 2 **[35]	**USA 3 **[87]	**Greece (Multiplex) **[50]	**Greece (Simplex) **[50]	**Australia **[52]	**Italy (Multiplex) **[53]	**Italy (Simplex) **[53]	**Canada **[54]

AITD	11.11	0.98	2.4	4.6	0.88			21.7	16	20	14.7	3.9	4.81	5.50		13,28							
Hyperthyroidism^a^						4.2									3.75		22.1	9.42	8.2	14.15			
Hypothyroidism ^a^						7.38												1.71	0.74	9.43			
T1D			13.5			7.53	78.9	39.7		12.53	30.9	14.49				74	93.2	14.41	5.41	49.65	11.70	3.51	1.93
RA				0.9		7.41	22.5	16.98	64	28.57					2.0	7.8	18.7	0.68	0.18	20.75			1.73
PA			2.14			12.38																	
SLE	462		13.35			5.71	312.5			595	163.4					390	225	42.8	25	471			
AdD						4.81																	
CD				3.6	0.54	0.39																	
VIT			0.8	2.75		1.6												0.86	0.09	4.72			
MS						7.61	86.2	130		164	67.6												
MG						9.13						9.17						68.49	7.09				
IBD				0.89		1.25	30	4.2			21.79				4.44	24		10.9	7.38	15.09			
PSO	14.8			1.6				10			15.69				2.67	25		5.48	1.77	33.9			
SS										7.9								6.85	1.77				
AS												2.29								56.6			
SSc									500	595										471			
IIM									400														

Table shows aggregation (λ) of ADs. The top row depicts the AD present in the proband while the first column shows the AD present in FDRs. The numbers correspond to the calculation of aggregation. ^a^Only autoimmune hypothyroidism and hyperthyroidism are depicted. Prevalences in the general population according to [2,29-32]. If a range was reported the mean was calculated. AdD, Addison's disease; AS, ankylosing spondylitis; AITD, autoimmune thyroid disease; CD, celiac disease; IBD, inflammatory bowel disease; IIM, idiopathic inflammatory myositis; Lith, Lithuania; MG, myasthenia gravis; MS, multiple sclerosis; NL, The Netherlands; PA, pernicious anemia; PSO, psoriasis; RA, rheumatoid arthritis; SLE, systemic lupus erythematosus; SS, Sjögren's syndrome; SSc, systemic sclerosis; Swed, Sweden; T1D, type 1 diabetes; VIT, vitiligo.

### Other autoimmune diseases

The systematic search we performed retrieved other studies that assessed familial autoimmunity besides the five ADs we were focused on. These ADs are SSc, SS, IBD, juvenile dermatomyositis (JDM), VIT, juvenile rheumatoid arthritis (JRA), juvenile SLE (JSLE), idiopathic inflammatory myositis (IIM), CD, and alopecia areata (AA).

### Systemic sclerosis

Four studies reported diagnosis of AITD, RA and SLE in FDRs of SSc probands [57-60]. Frech *et al*. [60] found a RR of 2.49 (95% C.I. 1.99 to 3.41) for familial autoimmunity in FDRs and a RR of 1.48 (95% C.I.1.34 to 2.39) for familial autoimmunity in second degree relatives.

### Sjögren´s syndrome

Two studies were found on this disease, one by Reveille *et al*. [61] and the other done by our group [62]. Both studies agreed on the occurrence of AITD and SLE among relatives. In addition to these ADs, we described the presence of RA [62] while Reveille *et al*. [61] reported the occurrence of MS and SSc.

### Inflammatory bowel disease

Two studies were retrieved. As mentioned earlier, Criswell *et al*. [35] found an increased frequency of familial autoimmunity among probands with IBD. A study conducted by Hemminki *et al*. [63] assessed familial autoimmunity within IBD probands. In UC patients when a parent had AS the SIR for UC in offspring was 1.6, for Crohn's disease (CrD) 2.5, for T1D 1.2, for Graves' disease 1.3, for MS 1.4, for polyarteritis nodosa (PAN) 2.0, for PSO 1.3, for RA 1.1, and for SLE 1.5 [63]. When singleton siblings had CD, the SIR for UC was 2.5, and for AS was 2.1. When a parent and a sibling had CrD the SIR for UC was 4.7 and for PSO was 4.3. In CrD patients, when a parent was diagnosed with UC, the SIR for CrD in offspring was 2.4, for AS was 1.8 and for PSO was 1.4. When singleton siblings had UC, the SIR was 2.8 and for AS was 2.1. When a parent and a sibling had UC, the SIR for CD was 5.0 and for RA was 2.2. In twins, the SIR for CrD-UC pairs was 4.9 [63].

### Vitiligo

For VIT, three studies assessed familial autoimmunity. The studies done by Alkhateeb *et al*. [64] and Laberge *et al*. [65] discovered a significant increase in the occurrence of three ADs other than VIT, namely, AITD, PA and AdD. Alkhateeb *et al*. also reported the occurrence of SLE, myasthenia gravis (MG) and SSc [64], while Laberge *et al*. found the presence of PSO and RA [65]. In Chinese patients, Zhang *et al*. [66] found a significant association with RA, AA and PSO.

### Juvenile rheumatoid arthritis

Two studies were found on familial autoimmunity in JRA [67,68]. Prahalad *et al*. [67] found that AITD accounted for the familial autoimmunity seen in these probands. Huang *et al*. [68] found, in addition to AITD, the presence of PSO, AS and SLE. Furthermore, Huang *et al*. [68] compared the prevalence of ADs in family members of probands with JRA against the prevalence in family members of probands with JSLE. Including all family members (that is, first, second and third degree relatives), JSLE probands had a greater prevalence of familial autoimmunity than probands with JRA. Nonetheless, in FDR the prevalence of ADs was not significantly different between these two diseases. Thus, familial autoimmunity is equally present in JRA and JSLE. Likewise, Pachman *et al*. [89] compared JRA to JDM and to healthy controls. The only statistically significant association was an increased frequency of RA and PA in FDR of JRA probands.

### Juvenile systemic lupus erythematosus

Two articles were found for this disease. While Huang *et al*. [68] found that 17% of the FDRs of JSLE probands were affected with an AD, Walters *et al*. [69] found a prevalence of 51%, with 35% of FDRs from JSLE probands having SLE, 30% AITD and 13% PSO.

### Idiopathic inflammatory myositis

Familial autoimmunity has also been assessed for IIM in two studies. The study by Ginn *et al*. [70] found that the most common disease was, once again, AITD followed by RA, T1D and PSO. In this article, OR for familial aggregation of ADs was calculated irrespective of disease status (that is, case or control). The strongest predictors were a blood relative and female gender. Niewold *et al*. [71] reported that FDRs of probands with JDM had a higher frequency of T1D or SLE than in FDRs of controls. However, this relationship did not reach statistical significance.

### Celiac disease

Three articles were found. Petaros et al. [72] found that the prevalence of familial autoimmunity was 4.9% among first and second degree relatives. The ADs that became manifest were AITD, PSO and T1D. In line with these results, Cataldo *et al*. [73] found an increased prevalence of ADs including AITD and T1D. Neuhausen *et al*. [74] also found a significant association with T1D and JRA. However, contrary to what was expected, they found a decreased prevalence of AITD.

### Alopecia areata

An increased frequency of AITD, VIT, T1D, PSO, and CD was found among FDRs of pediatric patients with AA [75].

## Discussion

**Figure 8:**
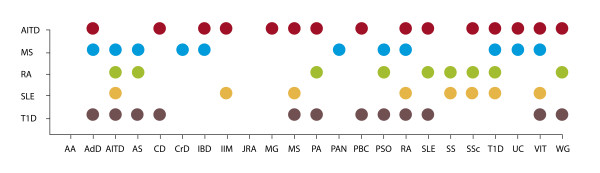
**Familial autoimmunity**. The vertical axis corresponds to the proband's disease and each disease individually. In the horizontal axis diseases present in first degree relatives are shown. Each color belongs to the proband's disease. The figure only includes significant results and may serve as a guide for clinical practice in order to search ADs in FDRs of probands. Note that familial autoimmune disease is excluded. AA, alopecia areata; AdD, Addison's disease; AS, ankylosing spondylitis; AITD, autoimmune thyroid disease; CD, celiac disease; CrD, Crohn's disease; FDR, first degree relative; IBD, inflammatory bowel disease; IIM, idiopathic inflammatory myositis; JDM, juvenile dermatomyositis; JRA, juvenile rheumatoid arthritis; JSLE, juvenile systemic lupus erythematosus; MAS, multiple autoimmune syndrome; MG, myasthenia gravis; MS, multiple sclerosis; PA, pernicious anemia; PAN, polyarteritis nodosa; PBC, primary biliary cirrhosis; PSO, psoriasis; RA, rheumatoid arthritis; SLE, systemic lupus erythematosus; SS, Sjögren's syndrome; SSc, systemic sclerosis; T1D, type 1 diabetes; UC, ulcerative colitis; VIT, vitiligo; WG, Wegener's granulomatosis. Note: Although AS is considered an auto inflammatory more than autoimmune disease [76] we show the results obtained.

The results found in this work support aggregation of diverse ADs (that is, familial autoimmunity) and the view of a common origin for ADs (that is, the autoimmune tautology). While polyautoimmunity [7-9,90,91] and familial autoimmune disease [1,9,42,92-94] are well-supported concepts in the medical literature, few articles have familial autoimmunity as their primary concern. Familial autoimmunity is still a topic that has not been thoroughly explored. To our knowledge, this is the first study specifically designed as a systematic review and meta-analysis to find evidence for familial autoimmunity in five major ADs. Familial autoimmunity uses the concept of 'autoimmune disease' as a trait that encompasses all pathologies showing evidence of an autoimmune origin. AITD followed by SLE and RA were the most frequent ADs encountered (Figure 8).

Our meta-analysis was developed in two stages. First, we wanted to determine the presence of familial autoimmunity as a trait in probands with the five index diseases mentioned earlier. However, a meta-analysis of studies having probands with SLE was not feasible. For the other four index diseases the meta-analyses indicate an increased risk of familial autoimmunity with RRs of 2.4, 1.6, 1.5, and 1.3 for AITD, T1D, RA and MS, respectively. It is not surprising to have AITD as the disease with a greater risk for familial autoimmunity as it is the most common AD worldwide. Meta-analyses with ORs as a measure of association were also done showing a significant relationship of familial autoimmunity with RA and MS probands.

Conversely, for our second approach, instead of grouping the studies for the meta-analyses by the proband's disease, we grouped the studies according to the disease present in FDRs. We must look at familial autoimmunity as a two way relationship depending upon which member of the nuclear family is the proband. Accordingly, we developed our second approach which also disclosed the presence of familial autoimmunity in a variety of ADs (Figure 4 and Additional file 1).

Several reasons may account for the heterogeneity found in our study, which have been also acknowledged by other authors [90,95], namely, different study designs, geographical differences, lack of adequate controls, use of a selected group of probands, and information bias, that is, recall bias [96], diverse population characteristics, and assorted study dates. The quality of studies was certainly influenced by the lack of awareness of familial autoimmunity. In addition, with time diagnostic approaches may have a better performance which may lead to a false increase in diagnoses frequencies.

Aggregation analyses disclosed extreme values (Table 3 and Table 4), with familial recurrence risk values over 100 as in the case of SSc (λ for SSc in FDRs of SLE probands = 500 to 595) or the case for SLE (λ for SLE in FDRs of MS probands = 471). In addition to these extreme values, we had conflicting results as in the case between MS and RA, and T1D and AITD for which some studies found a lack of aggregation whereas others found the opposite. These discrepancies may be explained by the fact that there are differences in prevalence according to geographical location, that aggregation involves genetic and environmental factors and, also, by the arbitrary calculation of means whenever a prevalence range was reported.

In the clinical setting, clinicians should be aware of familial autoimmunity whenever they are attending patients with ADs (Figure 8). A search for autoimmunity in their FDRs should be encouraged by exploring the presence of auto-antibodies [5] and other risk factors [20,21]. Since healthy subjects may have positive autoantibody titers, we decided only to include studies that were based on clinical diseases and not on the presence of autoantibodies.

ADs follow a multifactorial (or complex) inheritance pattern which represents an interaction between the collective effect of the genotype at multiple loci (polygenic or multigenic effects) either to raise or to lower susceptibility to disease, combined with a variety of environmental exposures that may trigger, accelerate, exacerbate, or protect against the disease process. Besides assessing the increased frequency of familial autoimmunity, the search also retrieved studies describing how this familial autoimmunity presents. A predominant inheritance of the autoimmunity trait from mothers was evident in some ADs including SS [62], juvenile idiopathic arthritis [97] and T1D [40]. This is indicative of a preferential transmission of susceptibility alleles from mothers to offspring. Maternal transmission of autoimmunity could be influenced by the high preponderance of ADs in women as compared with the general population. However, this higher than expected frequency of maternal transmission of the autoimmunity trait would warrant further studies of mitochondrial DNA, genomic imprinting, maternal-offspring compatibility, gene-environment and indirect genetic effects in ADs [62].

Another factor that influences familial autoimmunity is race [40,98,99]. Houghton *et al*. [98] compared the prevalence of familial autoimmunity between 'native' (Amerindian) and other groups in pediatric patients in the United States. In a small sample (6 Amerindians with SLE versus 34 non Amerindian population with SLE ), 83% of the native probands had a familial history of ADs while this was true for only 19% of the non-natives [98]. Meanwhile, with a larger sample size, the GLADEL study found that mestizos had more familial autoimmunity than other racial groups [42]. In fact, ancestry influences the risk and outcome of autoimmunity [99].

We would like to acknowledge the limitations of our study. First, the search was focused on five principal ADs, but we identified articles with probands from other ADs. It is probable that the number of articles retrieved from these ADs is less than if a specific search was done for each of these diseases. Second, we recall the heterogeneity of the study [100,101]. Third, in our search we found articles that did not distinguish between the presence of autoantibodies and a clinical diagnosis of an AD. This also should be taken into account in future studies as the presence of autoantibodies may occur in healthy people. Nevertheless, as stated earlier, they may herald a later onset of a given AD and, therefore, it may be clinically important to follow up those individuals.

## Conclusions

The importance of familial autoimmunity has been shown [102]. AITD followed by SLE and RA are the most frequent ADs in familial autoimmunity. Although non-genetic factors may have an effect on familial aggregation, shared genetic factors, in fact, may be the more likely cause for this aggregation [9]. Genes with larger effects (higher penetrance) are related to Mendelian patterns of inheritance, whereas those with smaller effects (lower penetrance) are related rather to complex traits, such as ADs. Identification of such genes, predisposing and affecting the outcome of ADs, is a major challenge for the near future. Given the clinical and etiologic heterogeneity of ADs, understanding the relationship of genotype to phenotype is an extremely important goal for research aimed at gene identification. Thus, further studies of familial autoimmunity will help in increasing the knowledge about the common mechanisms of autoimmunity. Genomics and other related disciplines will offer the tools to accomplish this task, allowing us to predict and prevent ADs, tailor individual medical decisions, and provide personalized healthcare while facilitating the patients' participation in their treatment and eventual cure of their disease [103].

## Abbreviations

AA: alopecia areata; AdD: Addison's disease; AD: autoimmune disease; AITD: autoimmune thyroid disease; AS: ankylosing spondylitis; C&C: case control; CD: celiac disease; CI: confidence interval; CrD: Crohn's disease; DL: discoid lupus; FDRs: first degree relatives; GLADEL: Grupo Latinoamericano de Estudio de Lupus; HA: hemolytic anemia; IBD: inflammatory bowel disease; IIM: idiopathic inflammatory myositis; JDM: juvenile dermatomyositis; JRA: juvenile rheumatoid arthritis;JSLE: juvenile systemic lupus erythematosus; MAS: multiple autoimmune syndrome; MG: myasthenia gravis; MS: multiple sclerosis; OR: odds ratio; PA: pernicious anemia; PAN: polyarteritis nodosa; PBC: primary biliary cirrhosis; PSO: psoriasis; RA: rheumatoid arthritis; RR: risk ratio; SIR: standardized incidence ratio; SLE: systemic lupus erythematosus; SS: Sjögren's syndrome; SSc: systemic sclerosis;T1D: type 1 diabetes; UC: ulcerative colitis; VIT: vitiligo; WG: Wegener's granulomatosis; λ, recurrence risk.

## Competing interests

The authors declare that they have no competing interests.

## Authors' contributions

JMA conceived the study, contributed to the literature search and drafted the article. JCR drafted the manuscript, did the literature search and carried out the data analysis. ARV helped to draft the manuscript, was the secondary reviewer for eligibility criteria and carried out the data analysis. All authors read and approved the final version of the manuscript.

## Pre-publication history

The pre-publication history for this paper can be accessed here:

http://www.biomedcentral.com/1741-7015/11/73/prepub

## Supplementary Material

Additional file 1 **Forest plots depicting risk ratios for familial autoimmunity in first degree relatives**. The figure shows two different analyses. From top to bottom: Addison's disease, autoimmune thyroid disease, ankylosing spondylitis, celiac disease, inflammatory bowel disease, discoid lupus, hemolytic anemia, inflammatory idiopathic myositis, immune thrombocytopenic purpura, localized scleroderma, pernicious anemia, myasthenia gravis, multiple sclerosis, polyarteritis nodosa, primary biliary cirrhosis, psoriasis, rheumatoid arthritis, systemic lupus erythematosus, Sjögren's syndrome, systemic sclerosis, type 1 diabetes, vitiligo, Wegener's granulomatosis. The summary effect (random effect model) is depicted as a diamond at the bottom of each analysis. The lateral points of each diamond indicate confidence intervals for this estimate. Click here for file

Additional file 2 **Funnel plots of the three meta-analyses showing publication bias**. The corresponding funnel plot shows the standard error on the Y axis and the log value for common effect size on the horizontal axis. From top to bottom: OR for Type 1 diabetes in first degree relatives, OR for multiple sclerosis in probands, RR for autoimmune thyroid disease in probands. Visual inspection of funnel plots suggested effect sizes for the mentioned analyses were scattered asymmetrically around a central effect. OR, odds ratio; RR, risk ratio. Click here for file
