# Supplementary material for: How do autoimmune diseases cluster in families? A systematic review and meta-analysis
Source: BMC Med. 2013 Mar 18;11:73. doi: 10.1186/1741-7015-11-73 (PMC3655934; doi:10.1186/1741-7015-11-73)
Supplement: Additional file 1 — Forest plots depicting risk ratios for familial autoimmunity in first degree relatives. The figure shows two different analyses. From top to bottom: Addison's disease, autoimmune thyroid disease, ankylosing spondylitis, celiac disease, inflammatory bowel disease, discoid lupus, hemolytic anemia, inflammatory idiopathic myositis, immune thrombocytopenic purpura, localized scleroderma, pernicious anemia, myasthenia gravis, multiple sclerosis, polyarteritis nodosa, primary biliary cirrhosis, psoriasis, rheumatoid arthritis, systemic lupus erythematosus, Sjögren's syndrome, systemic sclerosis, type 1 diabetes, vitiligo, Wegener's granulomatosis. The summary effect (random effect model) is depicted as a diamond at the bottom of each analysis. The lateral points of each diamond indicate confidence intervals for this estimate. [file 1741-7015-11-73-S1.PDF]

Forest plots depicting risk ratios for familial autoimmunity in FDRs

Addison’s disease

**Study name**	**Subgroup within study**	**Disease in FDR**	Risk ratio	Lower limit	Z-Value	Upper limit	p-Value
Boelaert 2010 (UK)13	AITD	AdD	5,970	1,585	2,641	22,480	0,008
Hemminki 2009 (Sweden)T1D1	T1D	AdD	2,400	1,445	3,384	3,985	0,001
Hemminki 2009 (Sweden)T1D2	T1D	AdD	3,910	1,496	2,782	10,219	0,005
Hemminki 2009 (Sweden)AITD10	AITD	AdD	3,060	1,055	2,059	8,873	0,039
Hemminki 2009 (Sweden)AITD9	AITD	AdD	2,520	1,227	2,517	5,176	0,012
Hemminki 2009 (Sweden)RA1	RA	AdD	1,050	0,383	0,095	2,878	0,924
Hemminki 2009 (Sweden)RA2	RA	AdD	0,520	0,055	-0,570	4,924	0,569
Nielsen 2008 (Denmark)	MS	AdD	3,300	1,254	2,419	8,683	0,016
			2,543	1,825	5,514	3,543	0,000

Risk ratio and 95% CI

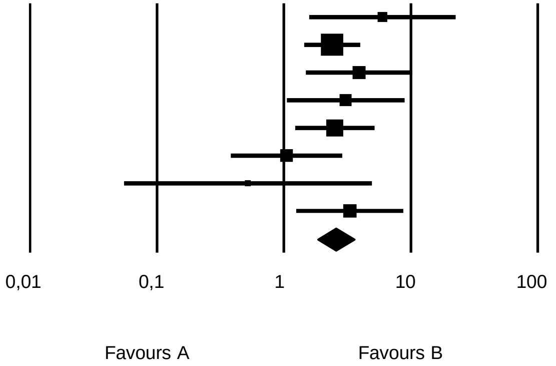

Meta Analysis

AITD

Study name	Subgroup within study	Disease in FDR	Risk ratio	Lower limit	Z-Value	Upper limit	p-Value
Hemminki 2009 (Sweden)T1D14	T1D	AITD	1,860	1,657	10,504	2,088	0,000
Hemminki 2009 (Sweden)T1D15	T1D	AITD	1,830	1,027	2,050	3,262	0,040
Hemminki 2009 (Sweden)T1D18	T1D	AITD	2,350	1,792	6,182	3,081	0,000
Hemminki 2009 (Sweden)T1D19	T1D	AITD	1,570	0,630	0,968	3,915	0,333
Hemminki 2009 (Sweden)RA17	RA	AITD	1,160	0,993	1,876	1,355	0,061
Hemminki 2009 (Sweden)RA18	RA	AITD	1,270	0,740	0,867	2,180	0,386
Hemminki 2009 (Sweden)RA20	RA	AITD	0,890	0,101	-0,105	7,810	0,916
Hemminki 2009 (Sweden)RA38	RA	AITD	1,710	0,784	1,347	3,732	0,178
Hemminki 2009 (Sweden)RA50	RA	AITD	1,540	1,216	3,585	1,950	0,000
Nielsen 2008 (Denmark)	MS	AITD	1,100	0,284	0,138	4,260	0,890
			1,604	1,298	4,378	1,982	0,000

Statistics for each study

Risk ratio and 95% CI

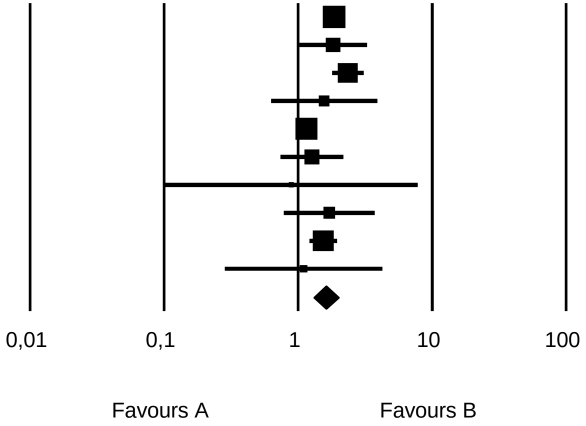

Meta Analysis

Ankylosing spondilitis

Study name	Subgroup within study	Disease in FDR	Risk ratio	Lower limit	Z-Value	Upper limit	p-Value
Hemminki 2009 (Sweden)T1D3	T1D	AS	1,380	1,022	2,105	1,863	0,035
Hemminki 2009 (Sweden)T1D4	T1D	AS	1,050	0,323	0,081	3,416	0,935
Hemminki 2009 (Sweden)AITD12	AITD	AS	1,160	0,677	0,540	1,988	0,589
Hemminki 2009 (Sweden)AITD13	AITD	AS	0,990	0,390	-0,021	2,516	0,983
Hemminki 2009 (Sweden)RA36	RA	AS	1,830	0,942	1,784	3,555	0,074
Hemminki 2009 (Sweden)RA60	RA	AS	2,960	2,150	6,648	4,076	0,000
Nielsen 2008 (Denmark)	MS	AS	1,300	0,637	0,721	2,654	0,471
			1,543	1,075	2,354	2,213	0,019

Statistics for each study

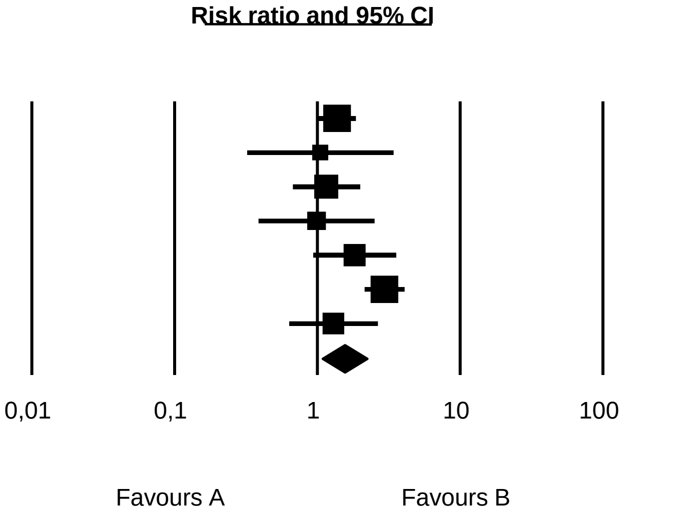

Meta Analysis

Celiac disease

Study name	Subgroup within study	Disease in FDR	Risk ratio	Lower limit	Z-Value	Upper limit	p-Value
Boelaert 2010 (UK)14	AITD	CD	5,730	3,403	6,566	9,648	0,000
Boelaert 2010 (UK)38	AITD	CD	6,100	1,623	2,677	22,928	0,007
Hemminki 2009 (Sweden)T1D6	T1D	CD	2,730	2,005	6,380	3,717	0,000
Hemminki 2009 (Sweden)T1D7	T1D	CD	1,920	1,132	2,420	3,256	0,016
Hemminki 2009 (Sweden)T1D8	T1D	CD	1,960	0,349	0,765	11,000	0,445
Hemminki 2009 (Sweden)AITD15	AITD	CD	1,100	0,512	0,244	2,364	0,807
Hemminki 2009 (Sweden)AITD16	AITD	CD	1,300	0,546	0,593	3,093	0,553
Hemminki 2009 (Sweden)RA5	RA	CD	1,250	0,679	0,717	2,300	0,473
Hemminki 2009 (Sweden)RA6	RA	CD	1,650	0,789	1,330	3,452	0,184
Nielsen 2008 (Denmark)	MS	CD	1,300	0,506	0,545	3,340	0,586
			2,060	1,415	3,770	2,999	0,000

Statistics for each study

Risk ratio and 95% CI

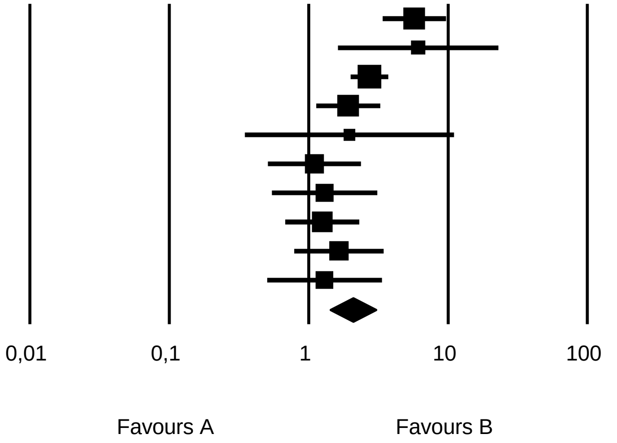

Meta Analysis

Inflammatory bowel disease

Study name	Subgroup within study	Disease in FDR	Risk ratio	Lower limit	Z-Value	Upper limit	p-Value
Boelaert 2010 (UK)17	AITD	IBD	2,340	1,647	4,745	3,325	0,000
Boelaert 2010 (UK)41	AITD	IBD	1,170	0,311	0,232	4,404	0,816
Hemminki 2009 (Sweden)T1D9	T1D	IBD	1,010	0,837	0,103	1,219	0,918
Hemminki 2009 (Sweden)T1D10	T1D	IBD	0,970	0,518	-0,095	1,817	0,924
Hemminki 2009 (Sweden)T1D46	T1D	IBD	1,230	1,063	2,775	1,424	0,006
Hemminki 2009 (Sweden)T1D47	T1D	IBD	1,080	0,605	0,261	1,927	0,794
Hemminki 2009 (Sweden)AITD18	AITD	IBD	0,950	0,662	-0,278	1,364	0,781
Hemminki 2009 (Sweden)AITD19	AITD	IBD	1,140	0,613	0,414	2,120	0,679
Hemminki 2009 (Sweden)AITD54	AITD	IBD	1,300	1,017	2,095	1,662	0,036
Hemminki 2009 (Sweden)AITD55	AITD	IBD	1,390	0,794	1,153	2,433	0,249
Hemminki 2009 (Sweden)RA34	RA	IBD	0,940	0,717	-0,448	1,232	0,654
Hemminki 2009 (Sweden)RA35	RA	IBD	1,310	0,773	1,004	2,219	0,315
Hemminki 2009 (Sweden)RA36	RA	IBD	1,350	0,241	0,342	7,557	0,733
Hemminki 2009 (Sweden)RA7	RA	IBD	0,930	0,655	-0,406	1,320	0,685
Hemminki 2009 (Sweden)RA8	RA	IBD	1,180	0,663	0,563	2,100	0,574
Nielsen 2008 (Denmark)	MS	IBD	1,400	1,036	2,189	1,892	0,029
Nielsen 2008 (Denmark)	MS	IBD	1,300	0,992	1,902	1,704	0,057
			1,197	1,070	3,130	1,340	0,002

Statistics for each study

Risk ratio and 95% CI

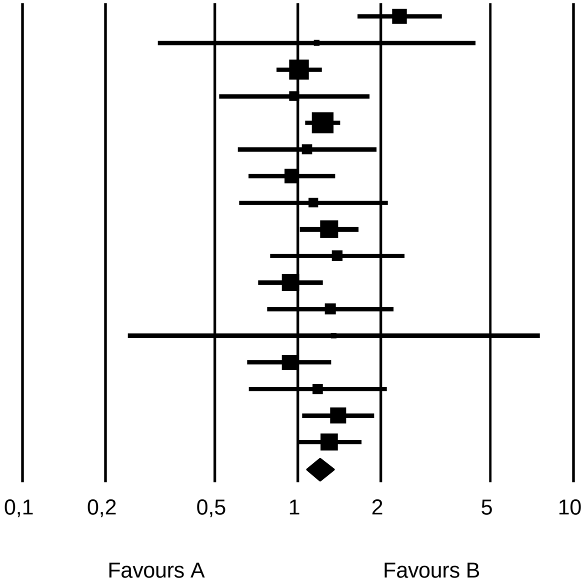

Meta Analysis

Discoid lupus

Study name	Subgroup within study	Disease in FDR	Risk ratio	Lower limit	Z-Value	Upper limit	p-Value
Hemminki 2009 (Sweden)T1D12	T1D	Discoid lupus	1,440	0,363	0,519	5,713	0,604
Hemminki 2009 (Sweden)AITD23	AITD	Discoid lupus	2,620	0,831	1,644	8,261	0,100
Hemminki 2009 (Sweden)AITD3	AITD	Discoid lupus	6,030	1,564	2,602	23,250	0,009
Hemminki 2009 (Sweden)RA15	RA	Discoid lupus	0,900	0,148	-0,114	5,474	0,909
Hemminki 2009 (Sweden)RA16	RA	Discoid lupus	1,940	0,347	0,755	10,842	0,450
			2,334	1,236	2,615	4,405	0,009

Statistics for each study

Risk ratio and 95% CI

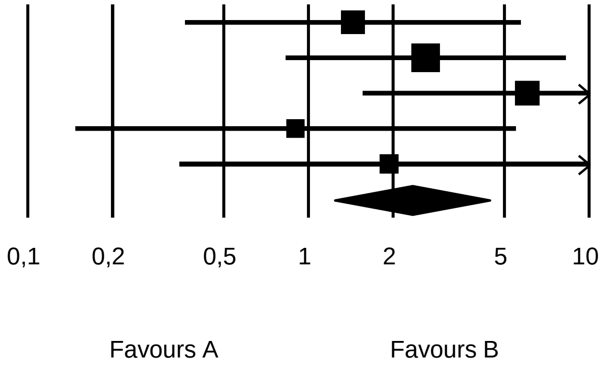

Meta Analysis

Hemolytic anemia

Study name	Subgroup within study	Disease in FDR	Statistics for each study					Risk ratio and 95% CI
			Risk ratio	Lower limit	Z-Value	Upper limit	p-Value	
Hemminki 2009 (Sweden)T1D5	T1D	HA	0,870	0,138	-0,148	5,502	0,882	
Hemminki 2009 (Sweden)AITD14	AITD	HA	1,760	0,647	1,108	4,785	0,268	
Hemminki 2009 (Sweden)RA4	RA	HA	1,920	0,937	1,783	3,933	0,075	
Nielsen 2008 (Denmark)	MS	HA	1,500	0,378	0,577	5,953	0,564	
			1,704	1,018	2,027	2,853	0,043	

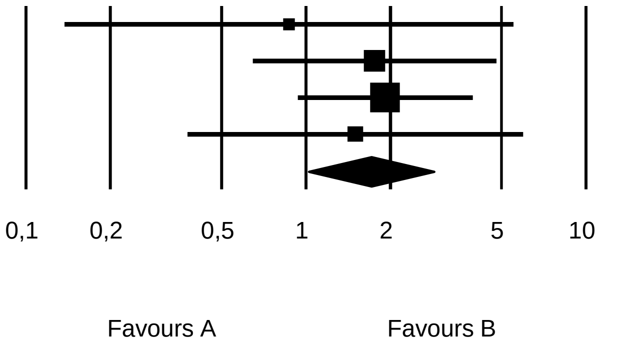

Meta Analysis

Inflammatory idiopathic myositis

Study name	Subgroup within study	Disease in FDRs	Risk ratio	Lower limit	Z-Value	Upper limit	p-Value
Hemminki 2009 (Sweden)T1D31	T1D	IIM	1,270	0,555	0,566	2,907	0,572
Hemminki 2009 (Sweden)AITD37	AITD	IIM	2,480	1,207	2,471	5,097	0,013
Hemminki 2009 (Sweden)AITD38	AITD	IIM	2,820	0,809	1,627	9,831	0,104
Hemminki 2009 (Sweden)RA30	RA	IIM	1,290	0,521	0,550	3,194	0,582
Hemminki 2009 (Sweden)RA31	RA	IIM	0,640	0,070	-0,396	5,839	0,692
			1,729	1,126	2,504	2,654	0,012

(Header over the statistics columns: **Statistics for each study**)

Risk ratio and 95% CI

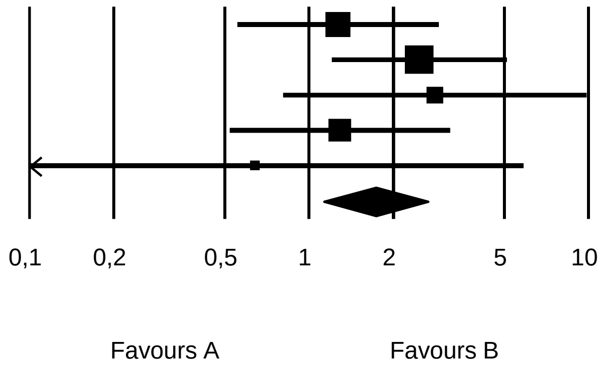

Meta Analysis

Immune thrombocytopenic purpura

Study name	Subgroup within study	Disease in FDRs	Statistics for each study: Risk ratio	Lower limit	Z-Value	Upper limit	p-Value
Hemminki 2009 (Sweden)T1D20	T1D	ITP	1,140	0,632	0,436	2,055	0,663
Hemminki 2009 (Sweden)T1D21	T1D	ITP	1,440	0,631	0,866	3,288	0,387
Hemminki 2009 (Sweden)AITD26	AITD	ITP	1,180	0,518	0,394	2,689	0,694
Hemminki 2009 (Sweden)AITD27	AITD	ITP	1,830	0,633	1,116	5,290	0,264
Hemminki 2009 (Sweden)RA21	RA	ITP	1,380	0,702	0,934	2,713	0,350
Hemminki 2009 (Sweden)RA22	RA	ITP	1,440	0,496	0,670	4,182	0,503
			1,321	0,959	1,704	1,818	0,088

Risk ratio and 95% CI

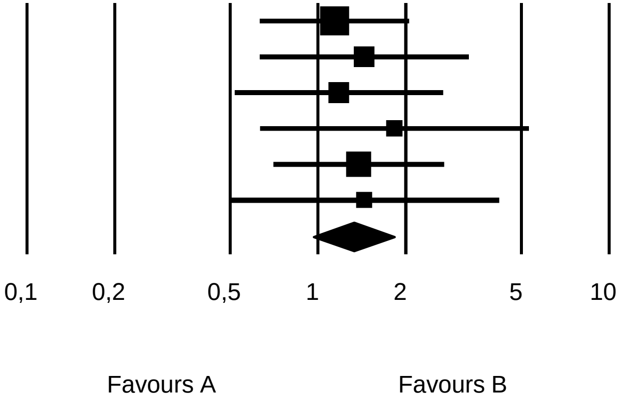

Meta Analysis

Localized ssc

Study name	Subgroup within study	Disease in FDRs	Risk ratio	Lower limit	Z-Value	Upper limit	p-Value
Hemminki 2009 (Sweden)T1D23	T1D	Localized SSc	1,330	0,335	0,405	5,287	0,685
Hemminki 2009 (Sweden)T1D24	T1D	Localized SSc	3,510	0,394	1,125	31,266	0,260
Hemminki 2009 (Sweden)AITD28	AITD	Localized SSc	1,190	0,298	0,246	4,760	0,806
Hemminki 2009 (Sweden)AITD4	AITD	Localized SSc	6,620	1,716	2,744	25,535	0,006
Hemminki 2009 (Sweden)RA39	RA	Localized SSc	2,200	0,391	0,894	12,385	0,371
Hemminki 2009 (Sweden)RA51	RA	Localized SSc	2,400	1,217	2,528	4,731	0,011
			2,362	1,459	3,498	3,825	0,000

Risk ratio and 95% CI

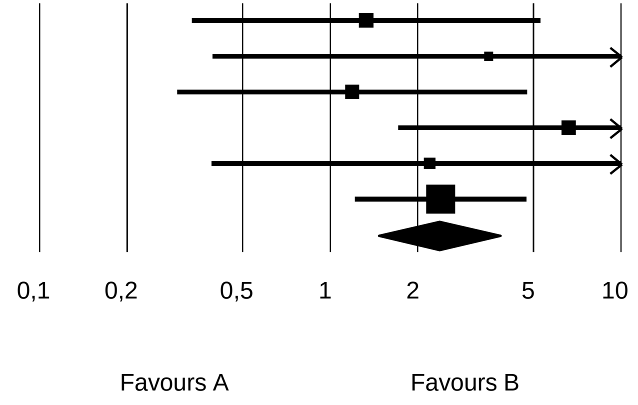

Meta Analysis

Pernicious anemia

Study name	Subgroup within study	Disease in FDRs	Risk ratio	Lower limit	Z-Value	Upper limit	p-Value
Boelaert 2010 (UK)20	AITD	PA	14,100	11,577	26,302	17,173	0,000
Boelaert 2010 (UK)44	AITD	PA	15,540	9,819	11,712	24,594	0,000
Hemminki 2009 (Sweden)T1D29	T1D	PA	3,090	2,044	5,352	4,671	0,000
Hemminki 2009 (Sweden)AITD7	AITD	PA	1,820	1,333	3,771	2,485	0,000
Hemminki 2009 (Sweden)RA41	RA	PA	1,560	0,405	0,646	6,013	0,518
Hemminki 2009 (Sweden)RA52	RA	PA	1,530	1,218	3,652	1,922	0,000
Nielsen 2008 (Denmark)	MS	PA	1,300	0,315	0,363	5,360	0,717
			3,443	1,344	2,576	8,818	0,010

Statistics for each study

Risk ratio and 95% CI

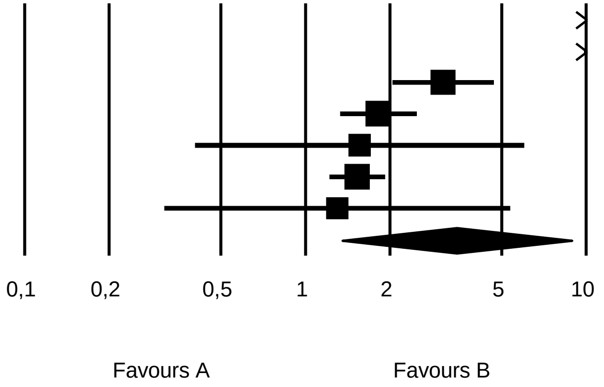

Meta Analysis

Myasthenia Gravis

Study name	Subgroup within study	Disease in FDRs	Risk ratio	Lower limit	Z-Value	Upper limit	p-Value
Boelaert 2010 (UK)18	AITD	MG	2,390	0,438	1,007	13,038	0,314
Hemminki 2009 (Sweden)T1D27	T1D	MG	1,180	0,657	0,555	2,118	0,579
Hemminki 2009 (Sweden)AITD32	AITD	MG	2,040	1,160	2,477	3,586	0,013
Hemminki 2009 (Sweden)AITD33	AITD	MG	1,590	0,453	0,724	5,582	0,469
Hemminki 2009 (Sweden)RA26	RA	MG	1,240	0,654	0,659	2,352	0,510
Hemminki 2009 (Sweden)RA27	RA	MG	2,020	0,751	1,392	5,435	0,164
Nielsen 2008 (Denmark)	MS	MG	2,000	0,816	1,516	4,899	0,129
			1,587	1,185	3,103	2,124	0,002

Statistics for each study

Risk ratio and 95% CI

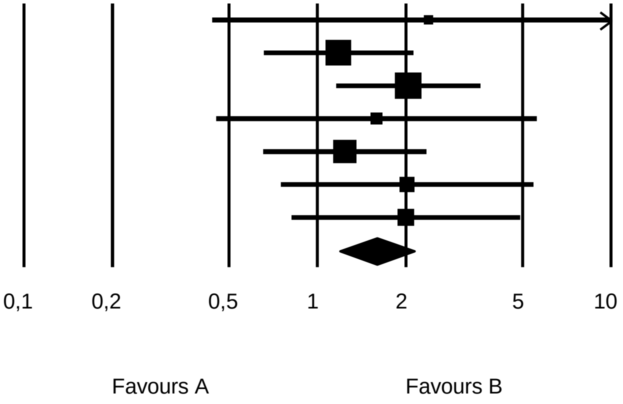

Meta Analysis

Multiple sclerosis

Study name	Subgroup within study	Outcome	Risk ratio	Lower limit	Z-Value	Upper limit	p-Value
Boelaert 2010 (UK)19	AITD	MS	3,450	1,323	2,532	8,998	0,011
Boelaert 2010 (UK)43	AITD	MS	3,110	1,195	2,324	8,097	0,020
Hemminki 2009 (Sweden)T1D25	T1D	MS	1,000	0,773	0,000	1,294	1,000
Hemminki 2009 (Sweden)T1D26	T1D	MS	1,240	0,556	0,525	2,768	0,599
Hemminki 2009 (Sweden)AITD29	AITD	MS	1,220	0,858	1,106	1,736	0,269
Hemminki 2009 (Sweden)AITD30	AITD	MS	1,210	0,588	0,517	2,492	0,605
Hemminki 2009 (Sweden)RA23	RA	MS	0,800	0,533	-1,079	1,200	0,281
Hemminki 2009 (Sweden)RA24	RA	MS	1,100	0,574	0,288	2,106	0,774
			1,205	0,927	1,395	1,566	0,163

Risk ratio and 95% CI

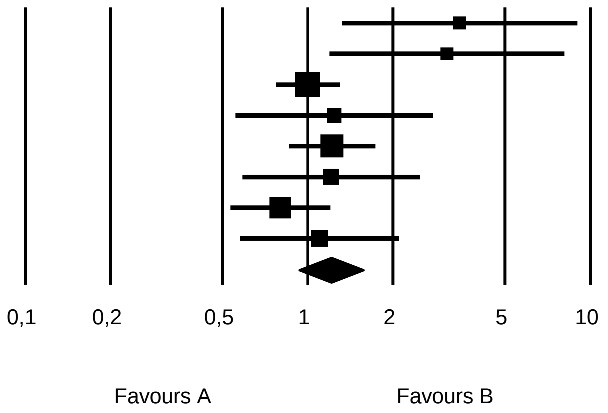

Meta Analysis

Polyarteritis nodosa

Study name	Subgroup within study	Outcome	Risk ratio	Lower limit	Z-Value	Upper limit	p-Value
Hemminki 2009 (Sweden)T1D30	T1D	PAN	1,400	0,512	0,656	3,828	0,512
Hemminki 2009 (Sweden)AITD35	AITD	PAN	1,600	0,651	1,025	3,930	0,305
Hemminki 2009 (Sweden)RA28	RA	PAN	1,730	0,877	1,582	3,412	0,114
Hemminki 2009 (Sweden)RA29	RA	PAN	2,200	0,491	1,030	9,857	0,303
Nielsen 2008 (Denmark)	MS	PAN	3,600	1,347	2,554	9,621	0,011
			1,903	1,259	3,056	2,875	0,002

Statistics for each study

Risk ratio and 95% CI

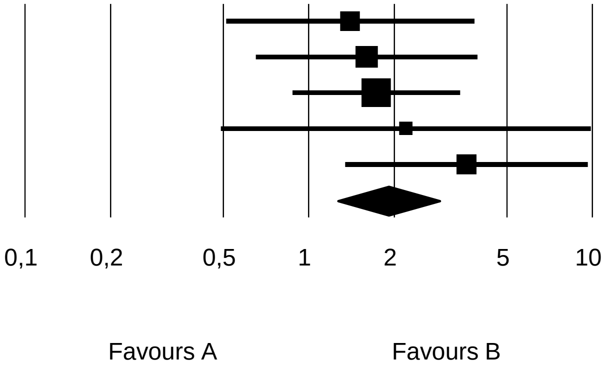

Meta Analysis

Primary biliary cirrhosis

Study name	Subgroup within study	Disease in FDRs	Statistics for each study				
			Risk ratio	Lower limit	Upper limit	Z-Value	p-Value
Hemminki 2009 (Sweden)T1D33	T1D	PBC	3,630	2,106	6,256	4,642	0,000
Hemminki 2009 (Sweden)AITD39	AITD	PBC	0,930	0,294	2,941	-0,124	0,902
Hemminki 2009 (Sweden)RA32	RA	PBC	0,770	0,281	2,109	-0,508	0,611
Nielsen 2008 (Denmark)	MS	PBC	1,200	0,300	4,800	0,258	0,797
			1,449	0,590	3,558	0,808	0,419

Risk ratio and 95% CI

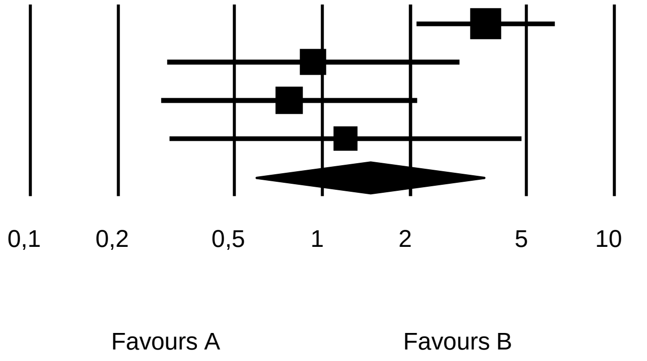

Meta Analysis

Psoriasis

Study name	Subgroup within study	Disease in FDRs	Risk ratio	Lower limit	Upper limit	Z-Value	p-Value
Hemminki 2009 (Sweden)T1D34	T1D	PSO	1,080	0,860	1,356	0,663	0,507
Hemminki 2009 (Sweden)T1D35	T1D	PSO	1,190	0,526	2,692	0,418	0,676
Hemminki 2009 (Sweden)AITD41	AITD	PSO	1,090	0,818	1,453	0,587	0,557
Hemminki 2009 (Sweden)AITD42	AITD	PSO	1,070	0,525	2,181	0,186	0,852
Hemminki 2009 (Sweden)RA53	RA	PSO	1,360	1,087	1,701	2,692	0,007
Hemminki 2009 (Sweden)RA56	RA	PSO	2,010	1,159	3,486	2,484	0,013
Nielsen 2008 (Denmark)	MS	PSO	0,700	0,452	1,084	-1,597	0,110
			1,151	0,945	1,401	1,395	0,163

(Statistics for each study)

Risk ratio and 95% CI

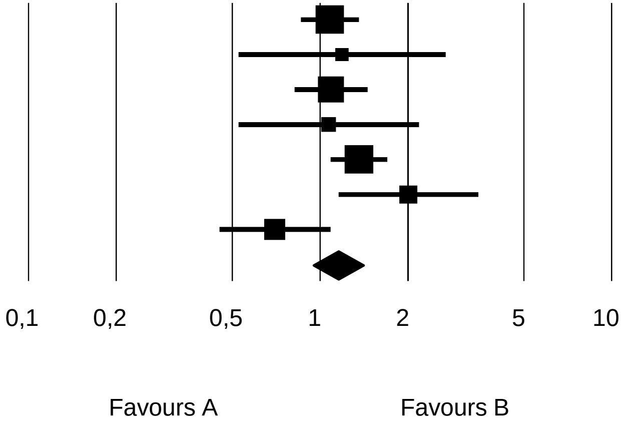

Meta Analysis

RA

Study name	Subgroup within study	Disease in FDRs	Risk ratio	Lower limit	Upper limit	p-Value
Boelaert 2010 (UK)21	AITD	RA	13,550	12,338	14,881	0,000
Boelaert 2010 (UK)45	AITD	RA	13,040	10,349	16,431	0,000
Hemminki 2009 (Sweden)T1D37	T1D	RA	2,120	1,902	2,363	0,000
Hemminki 2009 (Sweden)T1D38	T1D	RA	1,380	0,708	2,690	0,344
Hemminki 2009 (Sweden)AITD44	AITD	RA	1,380	0,765	2,488	0,284
Hemminki 2009 (Sweden)AITD8	AITD	RA	1,480	1,311	1,671	0,000
Nielsen 2008 (Denmark)	MS	RA	1,200	0,930	1,549	0,162
			2,811	1,141	6,924	0,025

Risk ratio and 95% CI

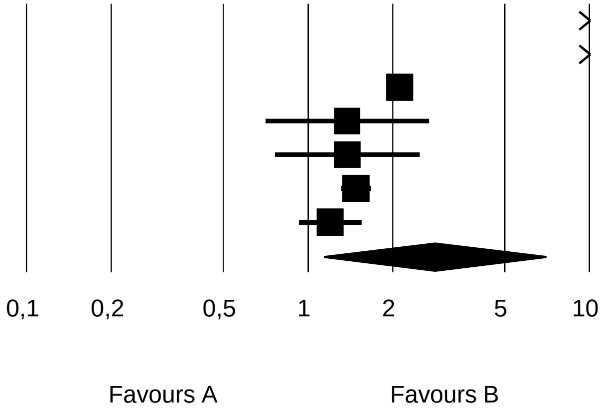

Meta Analysis

SLE

Study name	Subgroup within study	Disease in FDRs	Risk ratio	Lower limit	Upper limit	p-Value
Boelaert 2010 (UK)22	AITD	SLE	4,650	2,057	10,514	0,000
Boelaert 2010 (UK)46	AITD	SLE	7,480	2,022	27,671	0,003
Hemminki 2009 (Sweden)T1D42	T1D	SLE	2,045	1,500	2,788	0,000
Hemminki 2009 (Sweden)T1D43	T1D	SLE	1,420	0,528	3,816	0,487
Hemminki 2009 (Sweden)AITD49	AITD	SLE	1,840	0,770	4,399	0,170
Hemminki 2009 (Sweden)AITD50	AITD	SLE	1,400	0,865	2,266	0,171
Hemminki 2009 (Sweden)RA55	RA	SLE	2,130	1,542	2,942	0,000
Hemminki 2009 (Sweden)RA58	RA	SLE	2,770	1,399	5,485	0,003
Nielsen 2008 (Denmark)	MS	SLE	1,200	0,600	2,400	0,606
			2,066	1,594	2,679	0,000

Risk ratio and 95% CI

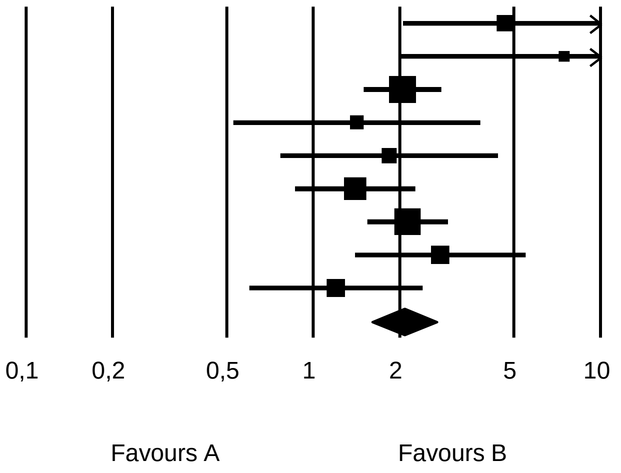

Meta Analysis

SS

Study name	Subgroup within study	Disease in FDRs	Risk ratio	Lower limit	Upper limit	p-Value
			Statistics for each study			
Hemminki 2009 (Sweden)T1D40	T1D	SS	0,830	0,303	2,273	0,717
Hemminki 2009 (Sweden)AITD47	AITD	SS	1,230	0,451	3,354	0,686
Hemminki 2009 (Sweden)AITD48	AITD	SS	1,010	0,117	8,739	0,993
Hemminki 2009 (Sweden)RA43	RA	SS	2,440	0,803	7,418	0,116
Hemminki 2009 (Sweden)RA57	RA	SS	2,250	1,181	4,287	0,014
Nielsen 2008 (Denmark)	MS	SS	1,700	0,694	4,164	0,246
			1,656	1,124	2,441	0,011

Risk ratio and 95% CI

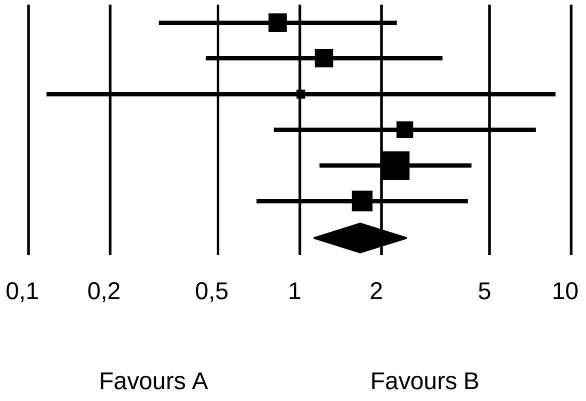

Meta Analysis

SSc

Study name	Subgroup within study	Outcome	Risk ratio	Lower limit	Upper limit	p-Value
Hemminki 2009 (Sweden)T1D44	T1D	SSc	0,810	0,478	1,372	0,433
Hemminki 2009 (Sweden)T1D45	T1D	SSc	0,740	0,213	2,575	0,636
Hemminki 2009 (Sweden)AITD52	AITD	SSc	1,340	0,818	2,195	0,245
Hemminki 2009 (Sweden)AITD53	AITD	SSc	1,370	0,492	3,817	0,547
Hemminki 2009 (Sweden)RA45	RA	SSc	1,990	0,893	4,434	0,092
Hemminki 2009 (Sweden)RA59	RA	SSc	1,650	1,149	2,369	0,007
Nielsen 2008 (Denmark)	MS	SSc	1,000	0,306	3,266	1,000
			1,307	1,012	1,688	0,040

(Statistics for each study)

Risk ratio and 95% CI

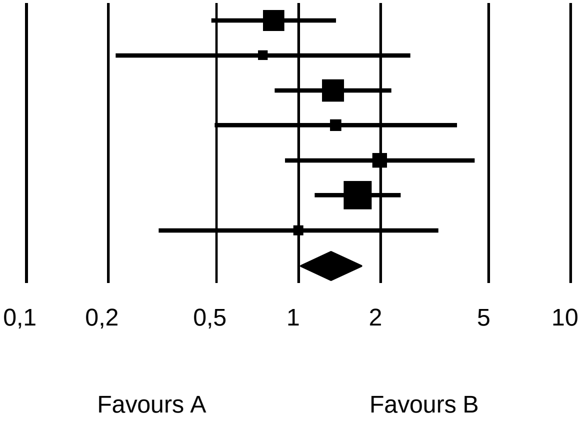

Meta Analysis

Type 1 Diabetes

Study name	Subgroup within study	Outcome	Risk ratio	Lower limit	Upper limit	p-Value
Boelaert 2010 (UK)23	AITD	T1D	3,640	2,858	4,636	0,000
Boelaert 2010 (UK)47	AITD	T1D	7,430	4,946	11,162	0,000
Hemminki 2009 (Sweden)AITD20	AITD	T1D	3,370	1,233	9,212	0,018
Hemminki 2009 (Sweden)AITD5	AITD	T1D	2,140	1,172	3,909	0,013
Hemminki 2009 (Sweden)RA11	RA	T1D	1,110	0,932	1,322	0,243
Hemminki 2009 (Sweden)RA12	RA	T1D	1,430	0,874	2,340	0,155
			2,573	1,298	5,100	0,007

Risk ratio and 95% CI

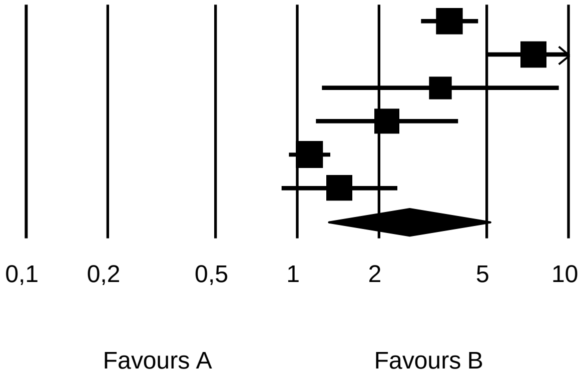

Meta Analysis

Vitiligo

Study name	Subgroup within study	Disease in FDRs	Risk ratio	Lower limit	Upper limit	p-Value
Boelaert 2010 (UK)24	AITD	VIT	6,090	4,291	8,643	0,000
Boelaert 2010 (UK)48	AITD	VIT	8,080	3,796	17,200	0,000
			6,402	4,660	8,796	0,000

Statistics for each study

Risk ratio and 95% CI

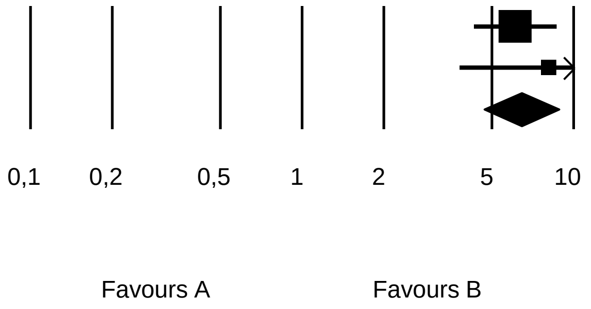

Meta Analysis

Wegener’s granulomatosis

Study name	Subgroup within study	Disease in FDRs	Statistics for each study			
			Risk ratio	Lower limit	Upper limit	p-Value
Hemminki 2009 (Sweden)T1D49	T1D	WG	2,120	1,424	3,156	0,000
Hemminki 2009 (Sweden)AITD57	AITD	WG	1,250	0,926	1,688	0,146
Hemminki 2009 (Sweden)AITD58	AITD	WG	1,310	0,293	5,852	0,724
Hemminki 2009 (Sweden)RA47	RA	WG	1,450	0,450	4,668	0,533
Hemminki 2009 (Sweden)RA54	RA	WG	1,340	1,096	1,639	0,004
Nielsen 2008 (Denmark)	MS	WG	0,500	0,083	3,000	0,448
			1,421	1,164	1,734	0,001

Risk ratio and 95% CI

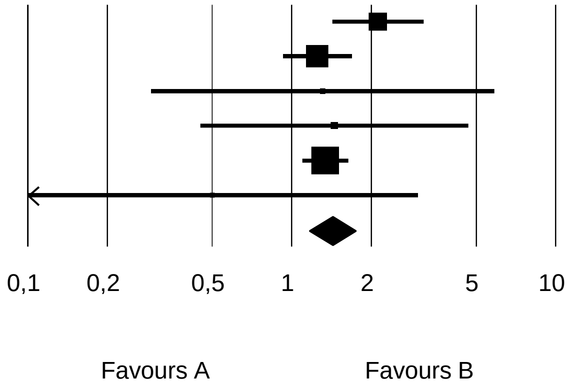

Meta Analysis
