# Supplementary material for: How do autoimmune diseases cluster in families? A systematic review and meta-analysis
Source: BMC Med. 2013 Mar 18;11:73. doi: 10.1186/1741-7015-11-73 (PMC3655934; doi:10.1186/1741-7015-11-73)

# Funnel plots

# Funnel plot: OR for T1D in first degree relatives

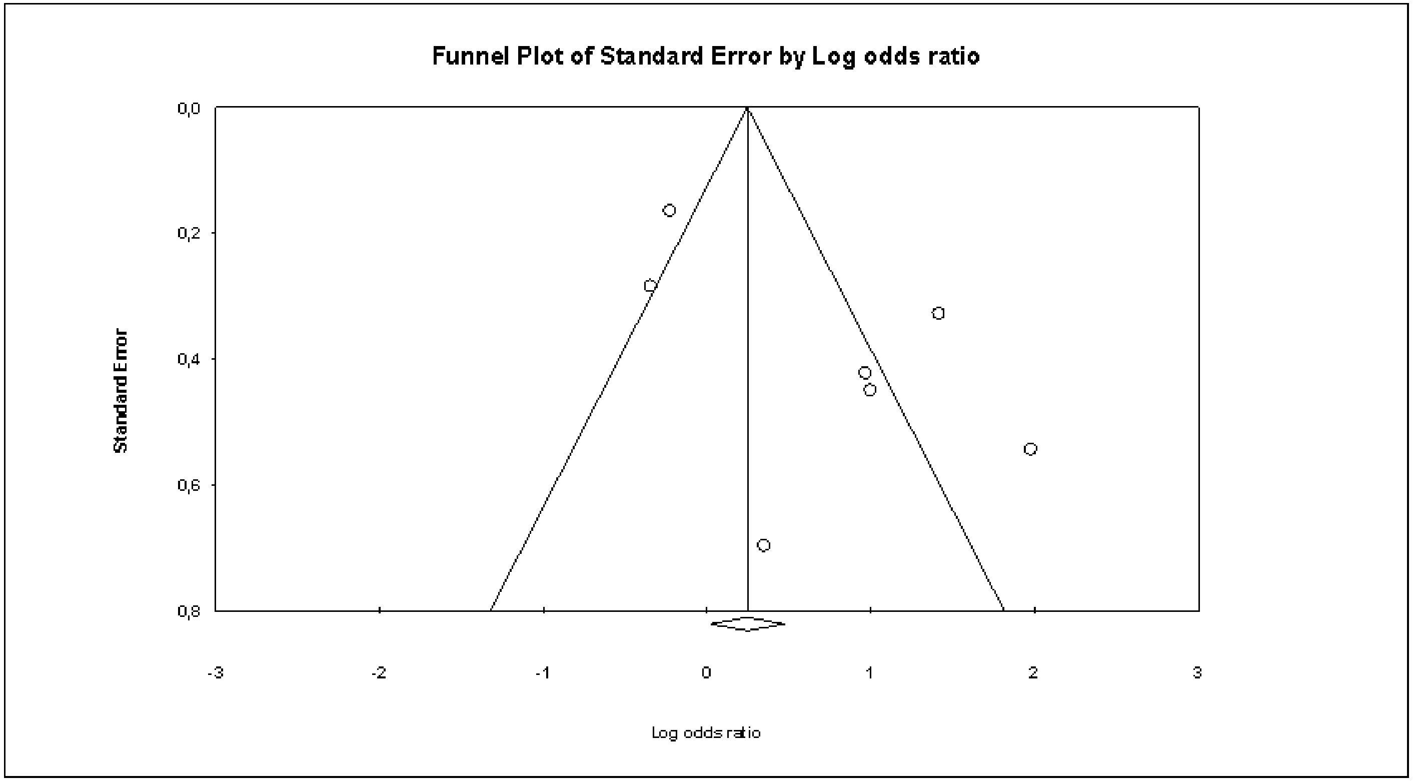

# Funnel plot: OR for MS in probands

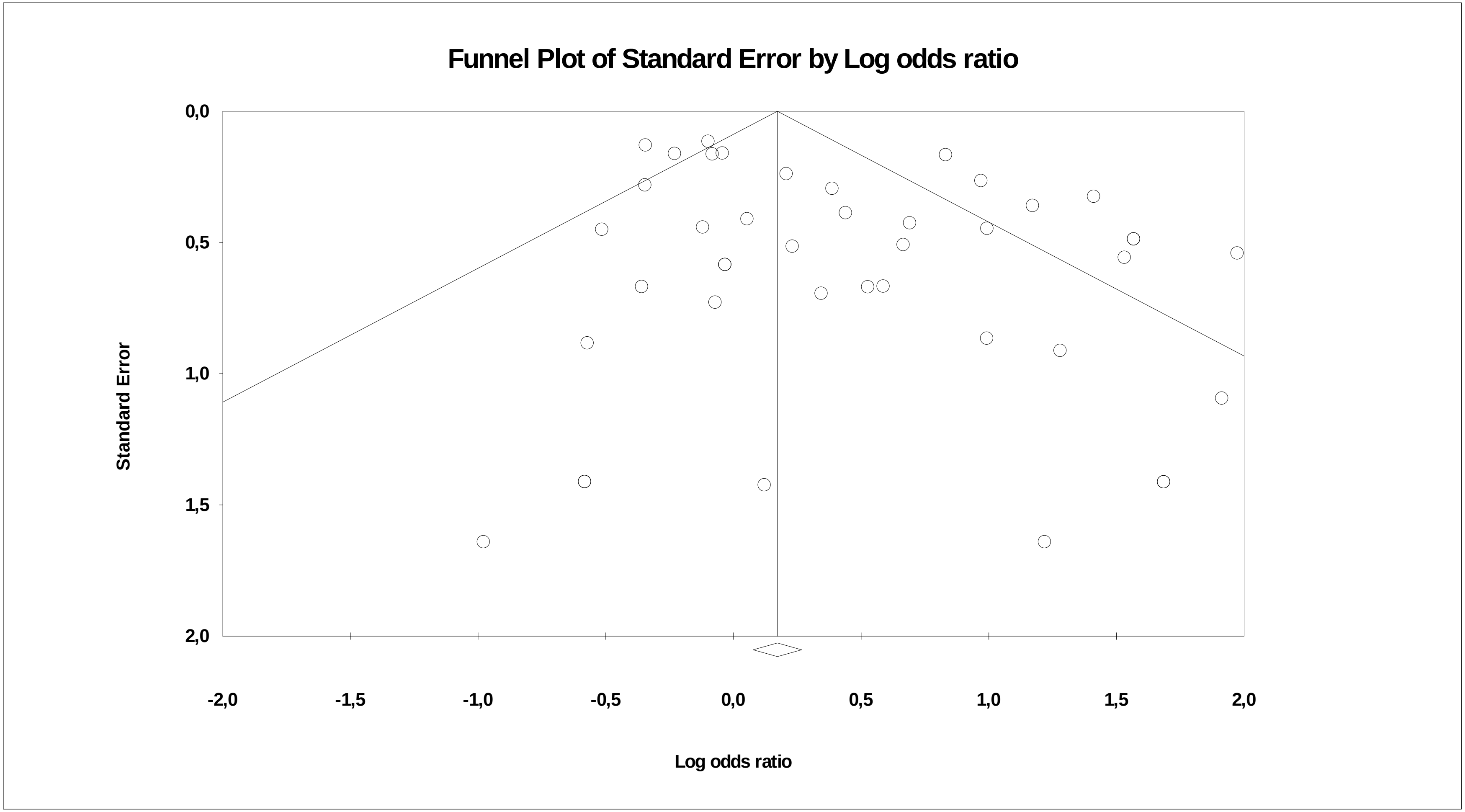

# Funnel plot: RR for AITD in probands

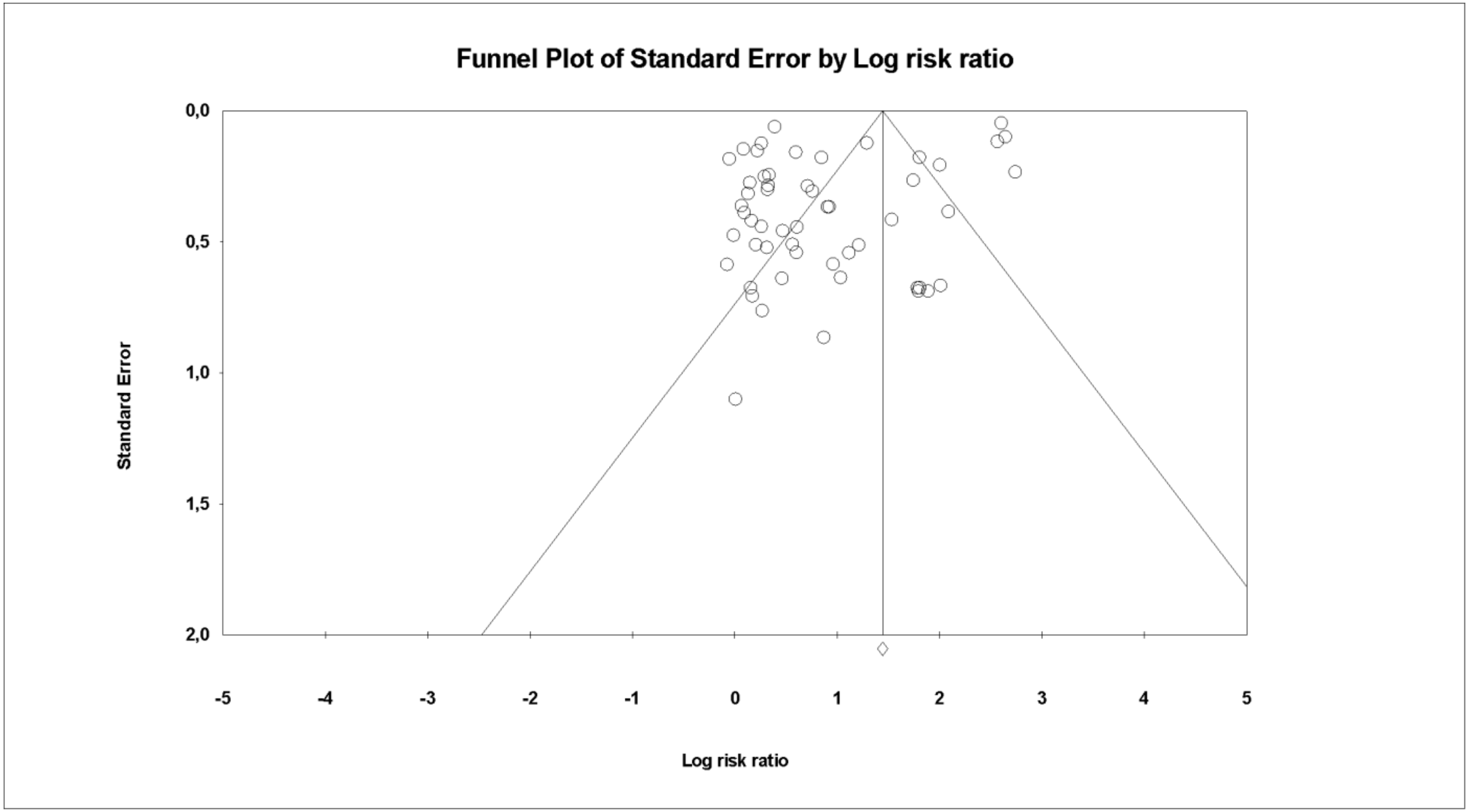

Supplement: Additional file 2 — Funnel plots of the three meta-analyses showing publication bias. The corresponding funnel plot shows the standard error on the Y axis and the log value for common effect size on the horizontal axis. From top to bottom: OR for Type 1 diabetes in first degree relatives, OR for multiple sclerosis in probands, RR for autoimmune thyroid disease in probands. Visual inspection of funnel plots suggested effect sizes for the mentioned analyses were scattered asymmetrically around a central effect. OR, odds ratio; RR, risk ratio. [file 1741-7015-11-73-S2.PDF]
